# Ovotransferrin and Its γ-Core-Containing Peptides Impair Pma1-Dependent Proton Homeostasis in *Candida albicans*

**DOI:** 10.3390/ijms27167415

**Published:** 2026-08-19

**Authors:** Victoria Antuña, Patricia Fierro, José F. Fierro, Ángel L. Álvarez, María T. Andrés

**Affiliations:** 1 Laboratory of Oral Microbiology (LMO), University Dental Clinic (CLUO), University of Oviedo, 33006 Oviedo, Asturias, Spain; uo282138@uniovi.es (V.A.); patricia.fierro@scsalud.es (P.F.); jffierro@uniovi.es (J.F.F.); 2 Health Research Institute of the Principality of Asturias (ISPA), 33011 Oviedo, Asturias, Spain; 3 Primary Care Emergency Service, Cantabrian Health Service, 39000 Santander, Cantabria, Spain; 4 Department of Functional Biology (Microbiology), Faculty of Medicine, University of Oviedo, 33006 Oviedo, Asturias, Spain; 5 Department of Biochemistry and Molecular Biology, University of Oviedo, 33006 Oviedo, Asturias, Spain; 6 SamerLabs S.L., Asturias Technology Park, 33428 Llanera, Asturias, Spain

**Keywords:** ovotransferrin, *Candida albicans*, γ-core peptides, Pma1, proton homeostasis, candidacidal activity, antifungal peptides

## Abstract

Ovotransferrin (OvoTf) is an iron-binding glycoprotein of avian innate immunity with antimicrobial activity, but its antifungal mechanism remains incompletely understood. We examined the candidacidal activity of OvoTf and two derived γ-core-containing peptides, kaliocin-3 (Kal-3) and kaliocin-4 (Kal-4), against *Candida albicans*. All three agents reduced fungal viability in a concentration-dependent manner, with minimal propidium iodide uptake, indicating a predominantly non-permeabilizing mechanism. Their activity was attenuated by extracellular Na^+^ and K^+^, whereas isoosmotic sorbitol did not confer protection. OvoTf induced partial K^+^ release, and tetraethylammonium increased cell survival. Inhibition of mitochondrial complex I or ATP synthase also reduced susceptibility, although none of the agents detectably suppressed oxygen consumption under the conditions tested. OvoTf, Kal-3, and Kal-4 caused plasma membrane depolarization, ATP accumulation, and impaired glucose-induced proton extrusion. This phenotype is consistent with disruption of Pma1-dependent proton and ion homeostasis, with mitochondrial bioenergetic activity contributing as a downstream requirement rather than constituting the primary target. Neither peptide caused detectable hemolysis; Kal-4 had minimal effects on mammalian cell metabolic viability, whereas Kal-3 reduced MCF-7 metabolic viability at higher concentrations. The activity of γ-core regions in both OvoTf lobes supports their further study as templates for mechanism-guided antifungal design.

## 1. Introduction

Ovotransferrin (OvoTf), formerly known as conalbumin, is an iron-binding glycoprotein of the transferrin family and one of the major protein components of avian egg white, where it accounts for approximately 12–13% of the total protein fraction [1,2]. Like other transferrins, OvoTf displays a conserved bilobal architecture specialized for reversible iron binding. However, transferrin-family proteins also perform functions that extend beyond metal transport, including roles in host defense, inflammatory regulation, and direct antimicrobial activity [2,3,4].

The antimicrobial activity of OvoTf has traditionally been attributed mainly to iron sequestration, which limits the availability of this essential metal to microorganisms [5,6]. Although biologically plausible, this explanation appears incomplete. Earlier studies showed that OvoTf retains antifungal activity against *Candida* spp. even after iron saturation. Together with the reported interaction of OvoTf with the *Candida* cell surface, this finding suggests that mechanisms other than iron sequestration contribute to its candidacidal effect [7,8]. More recently, OvoTf was also shown to affect bacterial membrane integrity under conditions mimicking the egg-white environment [9]. These findings suggest that OvoTf acts not only through nutritional immunity but also through direct antifungal mechanisms.

Defining this mechanism is relevant, as *Candida albicans*, although a common commensal member of the human mycobiota, remains a major opportunistic pathogen and has been recognized by the World Health Organization as a priority fungal pathogen [10,11,12]. This dual commensal–pathogen lifestyle, combined with the limited number of antifungal drug classes and the growing clinical relevance of antifungal tolerance and resistance, underscores the need to better understand host-derived mechanisms capable of compromising fungal viability. In this context, because opportunistic fungi such as *C. albicans* have evolved under continuous pressure from innate immune effectors, natural antifungal mechanisms may provide valuable templates for developing therapeutic strategies fundamentally different from conventional antifungal approaches and potentially less likely to select for adaptive resistance.

Interest in OvoTf has also increased because the protein contains embedded peptide regions with antimicrobial potential. Ibrahim and colleagues described OTAP-92, a large ovotransferrin-derived peptide with antibacterial activity and a membrane-damaging mechanism against bacteria [13,14]. Additional antimicrobial peptides derived from ovotransferrin and related transferrin proteins have subsequently been reported [15,16,17]. Previous work from our group and collaborators identified antimicrobial regions within transferrin-family proteins, including human lactoferrin (hLf) and ovotransferrin [17,18]. These regions include kaliocins, a group of transferrin-derived peptides containing γ-core motifs, conserved structural signatures widely associated with antimicrobial activity in cysteine-rich peptides and proteins [17].

The γ-core is an evolutionarily conserved multidimensional sequence–structure signature whose archetypal conformation comprises two antiparallel β-strands connected by a short turn. It commonly includes a GXC–X_3–9_–C pattern or an inverse CXG-containing variant and displays a characteristic cationic and amphipathic organization of charged and hydrophobic residues [18]. The γ-core framework has proved useful for identifying functionally important regions within diverse cysteine-stabilized antimicrobial peptides and proteins. Experimental studies have further shown that peptides encompassing complete γ-core regions can reproduce antimicrobial properties of their parent molecules. However, activity is not conferred by the GXC/CXG sequence consensus alone and may depend on the integrity of the motif, its charge and hydrophobicity, the contribution of flanking residues, and preservation of an appropriate conformational context [19,20,21,22]. More recent studies have exploited these principles to optimize γ-core-containing peptides and extend their antifungal potency or spectrum [22,23,24,25,26].

In contrast, the present study examines γ-core-containing regions embedded in two different lobes of the much larger bilobal ovotransferrin molecule. By testing kaliocin-3 (Kal-3) and kaliocin-4 (Kal-4) as isolated OvoTf-derived γ-core-containing peptides and comparing their cellular effects with those of the parental protein, this study investigates whether spatially separated γ-core-containing regions of a transferrin-family protein can retain a common non-permeabilizing antifungal phenotype.

Although the shared bilobal architecture and iron-binding capacity of ovotransferrin and human lactoferrin reflect their common membership in the transferrin family, these features alone do not necessarily predict similar antimicrobial mechanisms. A stronger rationale for their functional comparison is the presence of γ-core motifs in both proteins, a structural feature that Yount and Yeaman linked to antimicrobial activity across evolutionarily diverse host-defense proteins [18]. In *C. albicans*, human lactoferrin exerts in vitro candidacidal activity through a non-permeabilizing mechanism associated with impaired plasma membrane H^+^-ATPase Pma1 function, disruption of ion homeostasis, intracellular ATP accumulation, and mitochondrial participation in downstream regulated cell death [27,28,29]. Related non-permeabilizing mechanisms involving altered Pma1-dependent proton homeostasis have also been reported for human defensins and other cysteine-rich antimicrobial peptides and proteins harboring γ-core motifs [30,31]. Although these motifs should not be regarded as direct proof of Pma1 targeting, their recurrence among non-lytic host-defense molecules raises the possibility that γ-core-containing regions contribute to a broader antifungal strategy based on perturbation of fungal ion and proton homeostasis. This concept is particularly relevant for soluble transferrin-family proteins, in which embedded γ-core-containing regions may help explain why structurally related proteins such as lactoferrin and ovotransferrin could display convergent in vitro antifungal behavior [17,18].

Pma1, encoded by PMA1, is a fungal plasma-membrane single-subunit P-type H^+^-ATPase that extrudes protons across the plasma membrane at the expense of ATP hydrolysis, thereby contributing to cytosolic pH regulation and to the generation of the transmembrane electrochemical proton gradient that drives secondary nutrient transport [32,33,34]. Its essential role in fungal physiology, together with its divergence from human H^+^-ATPases, has made Pma1 an attractive target for selective antifungal development [35]. Accordingly, considerable effort has been directed toward the identification of new Pma1 inhibitors [36,37,38]. A simple cell-based phenotype characterized by plasma membrane depolarization, intracellular ATP accumulation, and defective glucose-induced extracellular acidification has been proposed as a functional signature of impaired Pma1-dependent proton extrusion and used as a screening approach to identify antifungal agents that inhibit Pma1 activity [37].

On this basis, we hypothesized that the candidacidal activity of OvoTf involves a non-permeabilizing mechanism associated with fungal proton and ion homeostasis, analogous to that previously described for human lactoferrin, and that γ-core-containing regions embedded within OvoTf may contribute to this activity. To test this hypothesis, we evaluated the antifungal activity and mechanism of OvoTf and two ovotransferrin-derived γ-core-containing peptides, kaliocin-3 (Kal-3) and kaliocin-4 (Kal-4), against *C. albicans*. Specifically, we asked whether these agents act through membrane damage or instead define a non-lytic antifungal mechanism centered on Pma1-dependent proton extrusion and ion-homeostatic failure.

## 2. Results

### 2.1. Structural Mapping Identifies Two γ-Core-Containing Regions in Ovotransferrin

**Figure 1:**
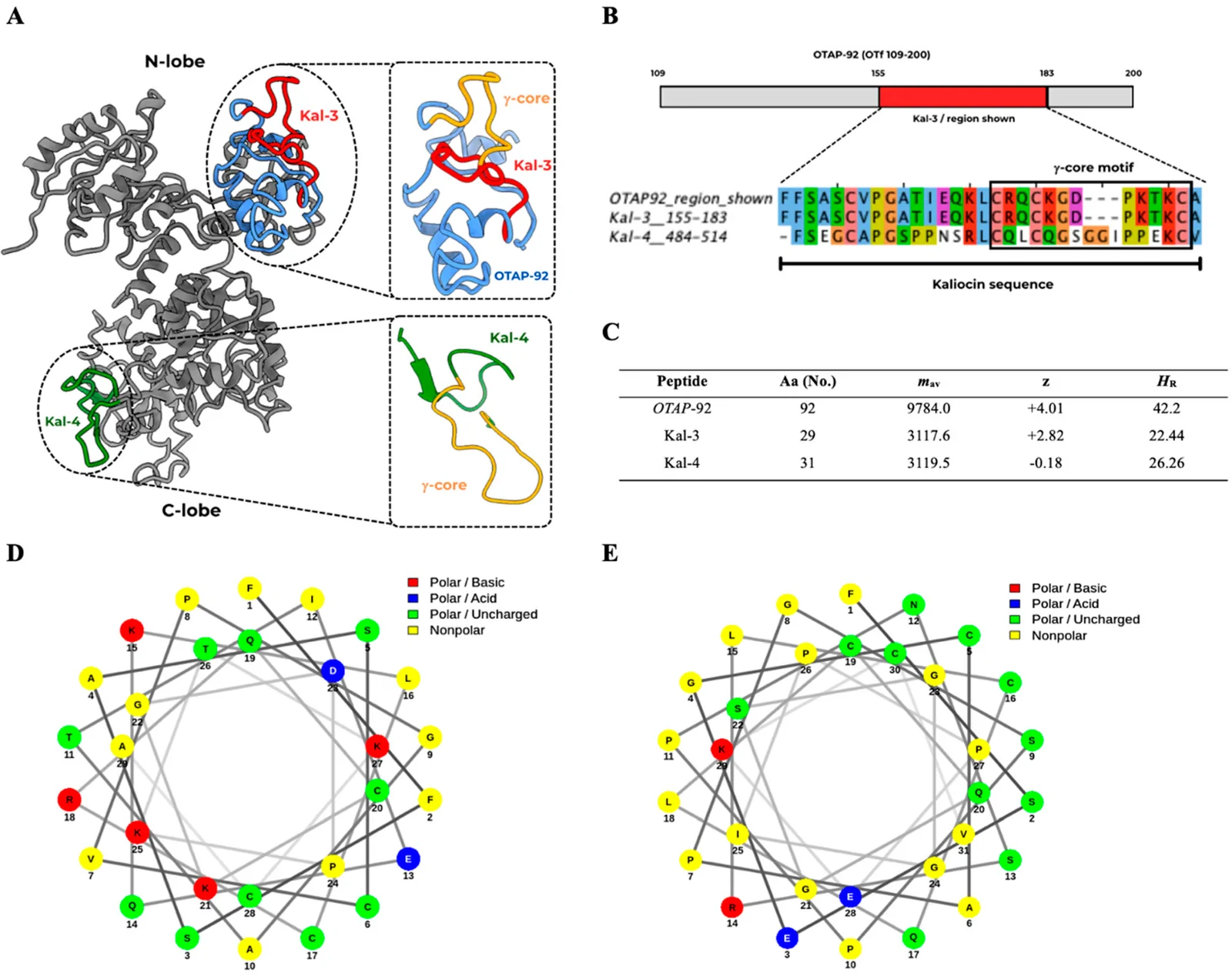
Structural, sequence, physicochemical, and amphipathic analysis of ovotransferrin-derived γ-core-containing peptides. (**A**) Three-dimensional representation of ovotransferrin (OvoTf) showing the N-lobe and C-lobe domains. In the N-lobe, the blue, red, and yellow regions together represent OTAP-92, with Kal-3 highlighted in red and its γ-core motif in yellow. Kal-4 is shown in green in the C-lobe, with its γ-core motif highlighted in yellow; the remaining ovotransferrin structure is shown in gray. Enlarged views depict the conformations of these regions within native OvoTf and should not be interpreted as experimentally determined structures of the isolated peptides. (**B**) Linear representation of OTAP-92 showing the position of Kal-3 within this larger antimicrobial fragment. The sequence comparison includes the OTAP-92 segment, Kal-3 (mature OvoTf residues 155–183), and Kal-4 (mature OvoTf residues 484–514). Residue numbering refers to the mature ovotransferrin chain; corresponding UniProt P02789 precursor coordinates are obtained by adding 19 residues. The γ-core-containing regions are boxed, alignment gaps are represented by dashes, and conserved or chemically related residues are color-coded. (**C**) Physicochemical properties of OTAP-92, Kal-3, and Kal-4, including peptide length, calculated average molecular mass (*m*_av_), calculated net charge (*z*) at pH 7.0, and relative hydrophobicity (*H*_R_). (**D**,**E**) Helical-wheel projections of Kal-3 (**D**) and Kal-4 (**E**), showing the spatial distribution of polar/basic, polar/acidic, polar/uncharged, and nonpolar residues according to the indicated color code. Residue numbers correspond to their position within each peptide sequence. Helical-wheel projections were generated using NetWheels.

We first performed an in silico analysis to determine whether ovotransferrin (OvoTf) contains discrete cysteine-associated regions with γ-core-like sequence features potentially compatible with antimicrobial activity. Structural mapping onto the three-dimensional fold of OvoTf identified two candidate regions, corresponding to kaliocin-3 (Kal-3) and kaliocin-4 (Kal-4), located in distinct regions of the bilobal transferrin architecture (Figure 1A). The Kal-3 and Kal-4 designations extend the previously established kaliocin nomenclature used for human lactoferrin-derived regions. Kal-3 was located within the N-lobe and overlapped with the previously described antimicrobial OTAP-92 region, whereas Kal-4 was located in the C-lobe. In the structural representation, both regions appeared at least partially exposed on the protein surface (Figure 1A), suggesting that they may be accessible for interactions with the fungal cell envelope.

To place the mapped OvoTf regions within the structural context of the transferrin family, their distribution was compared with previously described γ-core-containing regions in human lactoferrin (Figure S1). Both proteins displayed candidate regions in the N- and C-terminal lobes, although the corresponding sequences differed in charge, local environment, and degree of surface exposure. This comparison supports a shared capacity of the bilobal transferrin fold to harbor spatially separated antimicrobial regions, without implying structural or mechanistic identity of the isolated peptides.

Kal-3 corresponds to an internal segment of OTAP-92, which spans mature OvoTf residues 109–200. Specifically, Kal-3 maps to mature OvoTf residues 155–183, corresponding to positions 47–75 within OTAP-92 (Figure 1B). Kal-4, corresponding to mature OvoTf residues 484–514, also contains a related γ-core region, although it is located in a structurally distinct region of OvoTf within the C-lobe. The multiple sequence alignment showed that the OTAP-92 region represented in the figure encompasses Kal-3 and contains a cysteine-rich γ-core motif with the characteristic Cys-X-Gly (CXG) sequence pattern. Thus, Kal-3 can be interpreted as a γ-core-containing subregion embedded within the larger antimicrobial OTAP-92 peptide, whereas Kal-4 represents an additional OvoTf-derived γ-core-containing peptide located in the C-lobe.

The physicochemical properties of Kal-3 and Kal-4 were then compared with those of OTAP-92 (Figure 1C). OTAP-92 is a 92-residue peptide with a calculated average molecular mass of 9784.0 Da, a net charge of +4.01 at pH 7.0, and a relative hydrophobicity value, *H*_R_, of 42.2. By contrast, Kal-3 and Kal-4 are shorter peptides of 29 and 31 amino acids, respectively, with comparable molecular masses of approximately 3.1 kDa. Kal-3 displayed a calculated positive net charge of +2.82 and an *H*_R_ value of 22.44, whereas Kal-4 showed a near-neutral calculated net charge (−0.18) and an *H*_R_ value of 26.26.

Helical-wheel projections were next used to examine the spatial distribution of polar, charged, and nonpolar residues under an ideal α-helical projection model (Figure 1D,E). Kal-3 did not display a well-defined continuous hydrophobic face, despite its positive net charge. Kal-4 showed a more evident, but still incomplete, clustering of nonpolar residues, together with an overall near-neutral charge. Therefore, neither peptide showed the strongly segregated cationic–hydrophobic organization typically associated with classical lytic amphipathic α-helical antimicrobial peptides.

Together, these sequence, structural mapping, and physicochemical analyses indicate that OvoTf contains at least two regions compatible with the γ-core signature. One of them, Kal-3, is embedded within the previously described antimicrobial OTAP-92 fragment, suggesting that this γ-core-containing region could contribute to the antimicrobial properties previously attributed to the larger OvoTf-derived peptide OTAP-92. In contrast, Kal-4 represents an additional candidate γ-core region located in the C-lobe of OvoTf. These observations provided the rationale for evaluating Kal-3 and Kal-4 as independent γ-core-derived antifungal peptides in subsequent experiments.

### 2.2. Ovotransferrin and Its γ-Core-Containing Peptides Cause Concentration-Dependent Loss of Candida albicans Viability with Limited PI Uptake

**Figure 2:**
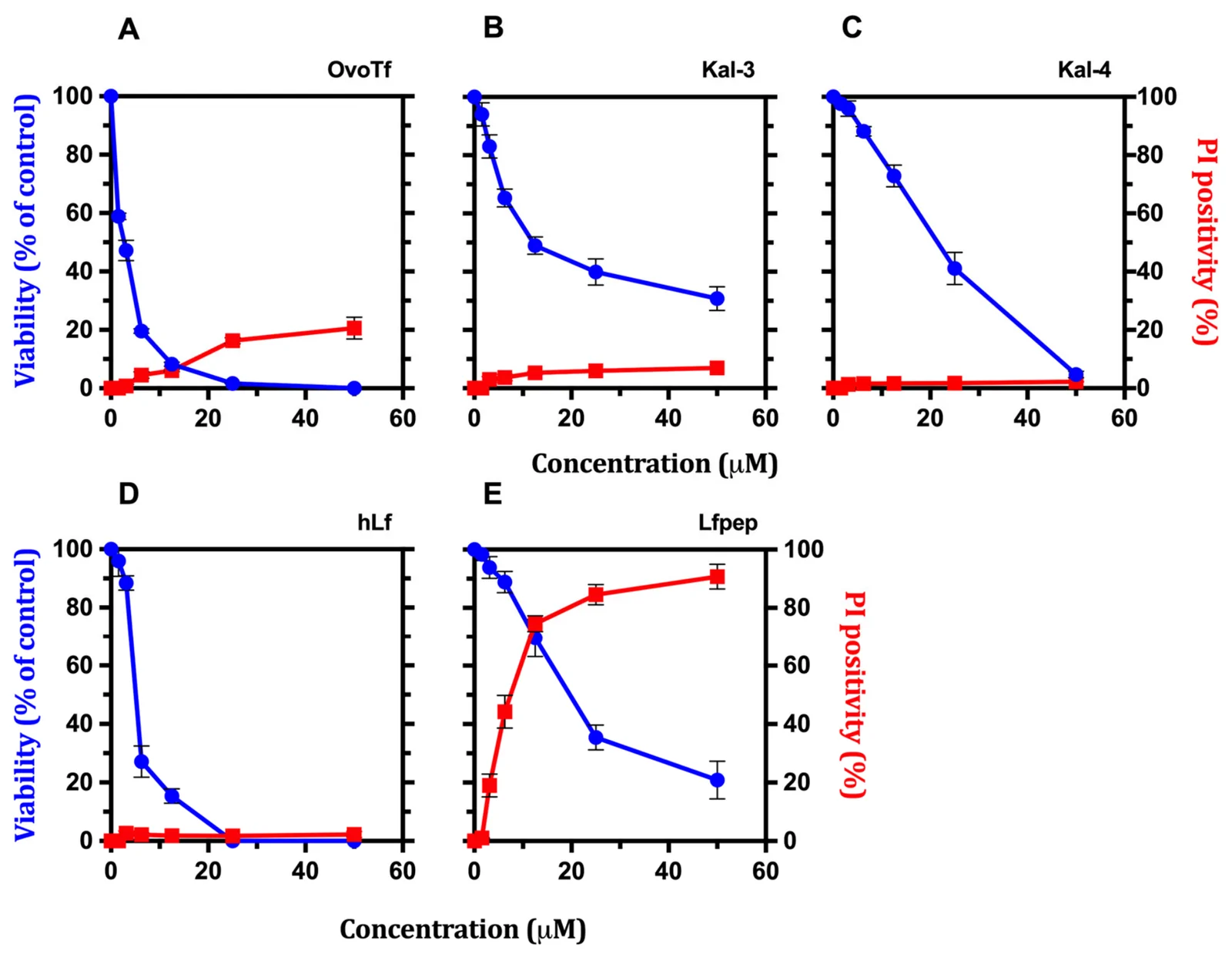
Comparative analysis of *Candida albicans* viability and PI uptake after treatment with ovotransferrin and derived peptides. *C. albicans* cells were incubated for 90 min at 37 °C in 10 mM Tris–HCl buffer, pH 7.4, with the indicated concentrations of (**A**) ovotransferrin (OvoTf), (**B**) kaliocin-3 (Kal-3), (**C**) kaliocin-4 (Kal-4), (**D**) human lactoferrin (hLf), or (**E**) the membrane-permeabilizing lactoferrin-derived peptide Lfpep. Cell viability was determined by CFU counting and expressed as a percentage of the untreated control. Plasma membrane permeabilization was assessed by propidium iodide (PI) uptake and expressed as the percentage of PI-positive cells. Blue circles represent viability, and red squares represent PI positivity. hLf and Lfpep were included as predominantly non-permeabilizing and membrane-permeabilizing reference controls, respectively. Data are shown as mean ± SD from three independent experiments. IC_50_ values were obtained by nonlinear regression of viability dose–response curves.

The candidacidal activity of ovotransferrin (OvoTf) and the OvoTf-derived γ-core-containing peptides kaliocin-3 (Kal-3) and kaliocin-4 (Kal-4) was evaluated against *C. albicans*. Cells suspended in 10 mM Tris–HCl buffer, pH 7.4 (Tris buffer), were incubated for 90 min at 37 °C with increasing concentrations of OvoTf, Kal-3 or Kal-4. All three agents caused a concentration-dependent loss of colony-forming ability, although they displayed distinct concentration–response profiles (Figure 2A–C). Nonlinear regression analysis of the dose–response curves using a four-parameter logistic model yielded IC_50_ values of 2.37 µM for OvoTf, 15.58 µM for Kal-3, and 19.56 µM for Kal-4. These values indicate that OvoTf displayed the highest antifungal potency among the ovotransferrin-based molecules tested.

To determine whether the reduction in viability was accompanied by loss of plasma membrane integrity, propidium iodide (PI) uptake was quantified in parallel under the same treatment conditions. A clear dissociation between loss of viability and PI uptake was observed. Kal-3 and Kal-4 markedly reduced viable cell counts, whereas PI positivity remained low throughout the tested concentration range. OvoTf also caused a pronounced loss of viability, but PI positivity increased only modestly at the highest concentrations and remained substantially below that produced by the membrane-permeabilizing control Lfpep (Figure 2). Thus, the reduction in colony-forming ability induced by OvoTf, Kal-3, and Kal-4 was not accompanied by a proportional increase in PI-detectable membrane permeabilization.

Human lactoferrin (hLf) and Lfpep were included as reference controls. hLf served as a predominantly non-permeabilizing candidacidal comparator, whereas Lfpep was used as a positive control for plasma membrane permeabilization. Consistent with their expected activities, hLf caused a marked reduction in viability with minimal PI uptake, with an IC_50_ value of 3.30 µM. In contrast, Lfpep reduced viability with an IC_50_ value of 19.50 µM and induced a concentration-dependent increase in PI-positive cells (Figure 2). The strong PI response elicited by Lfpep confirmed that substantial membrane permeabilization could be readily detected under the experimental conditions used.

The modest increase in PI positivity observed at the highest OvoTf concentrations may indicate limited membrane damage in a subpopulation of cells or secondary loss of membrane integrity after commitment to cell death. However, the endpoint nature of the assay does not establish whether this limited permeabilization precedes or follows the loss of viability.

Together, these findings show that OvoTf, Kal-3, and Kal-4 reduce *C. albicans* viability without a corresponding increase in substantial PI-detectable plasma membrane permeabilization. Their behavior more closely resembles that of hLf than that of the membrane-permeabilizing peptide Lfpep, supporting a predominantly non-permeabilizing mode of antifungal action under the conditions tested.

### 2.3. Ionic Conditions, but Not Non-Ionic Osmotic Stabilization, Attenuate Ovotransferrin- and γ-Core-Containing Peptide-Mediated Killing of Candida albicans

**Figure 3:**
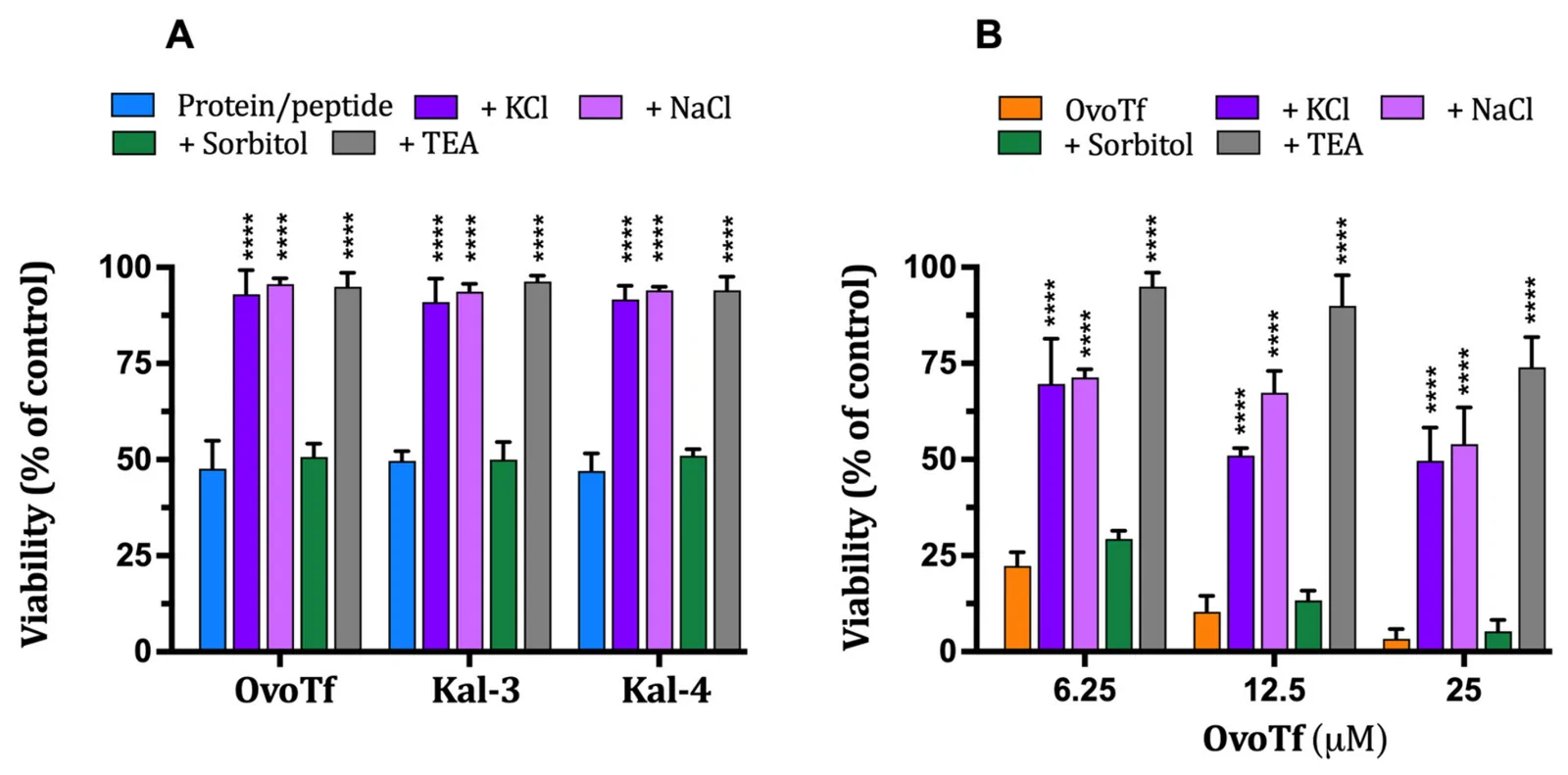
Effects of ionic and osmotic conditions on the candidacidal activity of ovotransferrin and its γ-core-containing peptides. *Candida albicans* cells were incubated for 90 min at 37 °C in 10 mM Tris–HCl buffer, pH 7.4. (**A**) Cells were exposed to OvoTf, Kal-3, or Kal-4 at their independently determined IC_50_ values. (**B**) Cells were exposed to OvoTf at 6.25, 12.5, or 25 µM. Treatments were performed alone or with KCl (150 mM), NaCl (140 mM), sorbitol (300 mOsm kg^−1^ H_2_O) or TEA (10 mM). Sorbitol was used as a non-ionic osmotic control. Viability was determined by CFU counting and expressed relative to the corresponding additive-matched untreated controls. Data are mean ± SD from three independent experiments. Statistics: two-way ANOVA with Dunnett’s test versus compound alone; **** *p* < 0.0001.

We next examined whether extracellular ionic conditions modulate the candidacidal activity of OvoTf and its γ-core-containing peptides. In an initial concentration screen, cells were exposed to OvoTf, Kal-3, or Kal-4 at their respective IC_50_ concentrations in Tris buffer supplemented with graded concentrations of KCl or NaCl ranging from 0 to 150 mM, at 10 mM intervals. Protection by either salt became apparent at concentrations of ≥30 mM and was maintained at higher concentrations. Accordingly, 150 mM KCl and 140 mM NaCl were selected as representative high-salt conditions for the comparative experiments shown in Figure 3. When OvoTf, Kal-3, and Kal-4 were tested at their respective IC_50_ concentrations, both salts markedly increased cell viability relative to the corresponding agent-only conditions (Figure 3A).

To determine whether this protective effect was maintained at OvoTf concentrations above its IC_50_, cells were additionally exposed to 6.25, 12.5, or 25 µM OvoTf (Figure 3B). KCl and NaCl increased cell viability at each OvoTf concentration tested, although the protection remained partial, particularly at the higher OvoTf concentrations. Thus, the salt-associated increase in viability was not limited to cells exposed to OvoTf at its IC_50_ concentration.

Because salt addition alters both ionic composition and osmolality, we next examined whether non-ionic osmotic stabilization alone could account for this protection. Candidacidal assays were therefore performed in the presence of sorbitol adjusted to 300 mOsm kg^−1^ H_2_O, providing a non-ionic osmotic condition comparable to those of the KCl- and NaCl-containing media. In contrast to both salts, sorbitol produced only minor changes in the viability of cells exposed to OvoTf, Kal-3, or Kal-4 and did not reproduce the marked protective effects of KCl or NaCl (Figure 3A). Similarly, sorbitol did not reproduce the protective effects of either salt across the three higher OvoTf concentrations examined (Figure 3B). These findings indicate that salt-mediated protection cannot be attributed solely to non-ionic osmotic stabilization.

In addition, in salt-free Tris buffer, exposure to OvoTf induced the extracellular release of K^+^, corresponding to approximately 24 ± 6% of the total cell-associated K^+^ content and therefore consistent with partial loss of intracellular K^+^. Such release could result either from limited membrane leakage through lesions insufficient to permit PI entry or from K^+^ efflux through endogenous transport pathways. To further examine whether K^+^ efflux contributes to OvoTf- and peptide-mediated killing, cells were pretreated with tetraethylammonium (TEA; 10 mM), a broad inhibitor of TEA-sensitive K^+^ transport processes, before exposure to OvoTf, Kal-3, or Kal-4. TEA markedly increased survival in cells exposed to each of the three agents (Figure 3A). TEA also increased viability at all three OvoTf concentrations examined in Figure 3B, although the magnitude of protection was lower at 25 µM OvoTf than at 6.25 or 12.5 µM. This pattern is consistent with previous findings obtained with hLf.

Taken together, these results show that KCl, NaCl, and TEA attenuate the candidacidal activity of OvoTf, Kal-3, and Kal-4 under conditions in which a non-ionic osmotic control does not reproduce the same degree of protection.

### 2.4. Mitochondrial Bioenergetic Activity Is Functionally Required for the Full Candidacidal Response

#### 2.4.1. Respiratory Activity Promotes Susceptibility to Ovotransferrin and Its Derived Peptides

*C. albicans* is generally considered a Crabtree-negative yeast [39] and, under the low-glucose, respiration-permissive conditions used in these assays, relies substantially on mitochondrial respiration for energy generation. Because previous studies showed that the antifungal activity of human lactoferrin (hLf) is influenced by the respiratory state of *C. albicans* [28], we asked whether mitochondrial electron transport also contributes to ovotransferrin (OvoTf)-induced killing.

**Figure 4:**
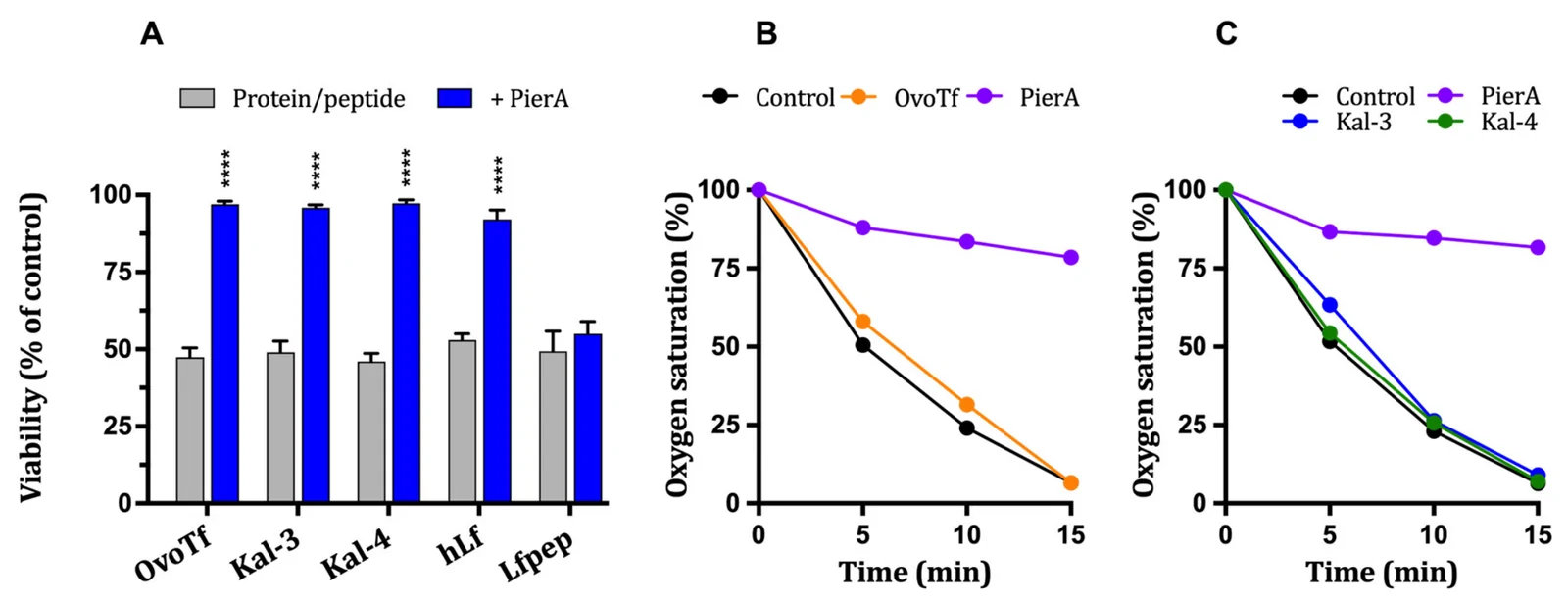
Effect of complex I inhibition on ovotransferrin- and peptide-induced killing and oxygen consumption in *Candida albicans*. (**A**) Cells were preincubated with piericidin A (PierA; 16 µM) and then exposed to OvoTf, Kal-3, Kal-4, hLf, or Lfpep at their respective IC_50_ concentrations. Viability was determined by CFU counting and expressed relative to untreated controls. Statistics: two-way ANOVA with Dunnett’s test vs. compound alone; **** *p* < 0.0001. (**B**,**C**) Oxygen consumption was monitored in untreated *C. albicans* cells and cells exposed to OvoTf (**B**), Kal-3 or Kal-4 (**C**). Piericidin A (PierA) was included as a positive control of respiratory inhibition. Data are mean ± SD from three independent experiments. Statistical significance versus the untreated control at each time point was determined by two-way repeated-measures ANOVA followed by Šídák’s multiple-comparisons test.

Cells were preincubated with piericidin A (PierA), an inhibitor of mitochondrial complex I–linked respiration, and then exposed to OvoTf, Kal-3, Kal-4, hLf, or Lfpep at their respective IC_50_ concentrations. Cells pretreated with PierA showed substantially higher survival after exposure to OvoTf, Kal-3, Kal-4, or hLf than cells treated with each antimicrobial agent alone (Figure 4A). In contrast, this protective effect was not observed with Lfpep, included as a membrane-permeabilizing control, whose candidacidal activity remained largely unaffected by PierA.

To assess whether OvoTf and its derived peptides affect mitochondrial respiration, oxygen consumption was monitored to determine whether OvoTf or the derived kaliocins directly inhibited cellular respiration. The percentage of oxygen remaining decreased progressively in untreated cells and followed a broadly similar time course in cells exposed to OvoTf, Kal-3, or Kal-4. OvoTf did not differ significantly from the untreated control at 5, 10, or 15 min. Kal-4 also showed no significant differences from the control at any time point. Kal-3 showed a modest difference at 5 min (*p* < 0.05), but this difference was no longer evident at 10 or 15 min. By contrast, piericidin A, included as a positive control for respiratory inhibition, significantly reduced oxygen consumption at all measured time points. At 15 min, the percentage of oxygen remaining was 6.7 ± 1.5% in control cells, 6.3 ± 0.6% with OvoTf, 9.0 ± 2.0% with Kal-3, 7.0 ± 1.7% with Kal-4, and 78.7 ± 2.5% with piericidin A. These results indicate that neither OvoTf nor the derived kaliocins caused sustained inhibition of cellular respiration under the conditions tested.

#### 2.4.2. Mitochondrial ATP Synthase Activity Is Required for the Full Ovotransferrin- and Peptide-Induced Candidacidal Response

Mitochondrial F_1_F_0_-ATP synthase activity has previously been implicated in the candidacidal response induced by human lactoferrin [28]. We therefore examined whether inhibition of mitochondrial ATP synthase altered the susceptibility of *C. albicans* to OvoTf and its derived peptides. Cells were preincubated with oligomycin A (16 μg/mL) for 15 min before exposure to OvoTf, Kal-3, Kal-4, or hLf at their respective IC_50_ concentrations.

**Figure 5:**
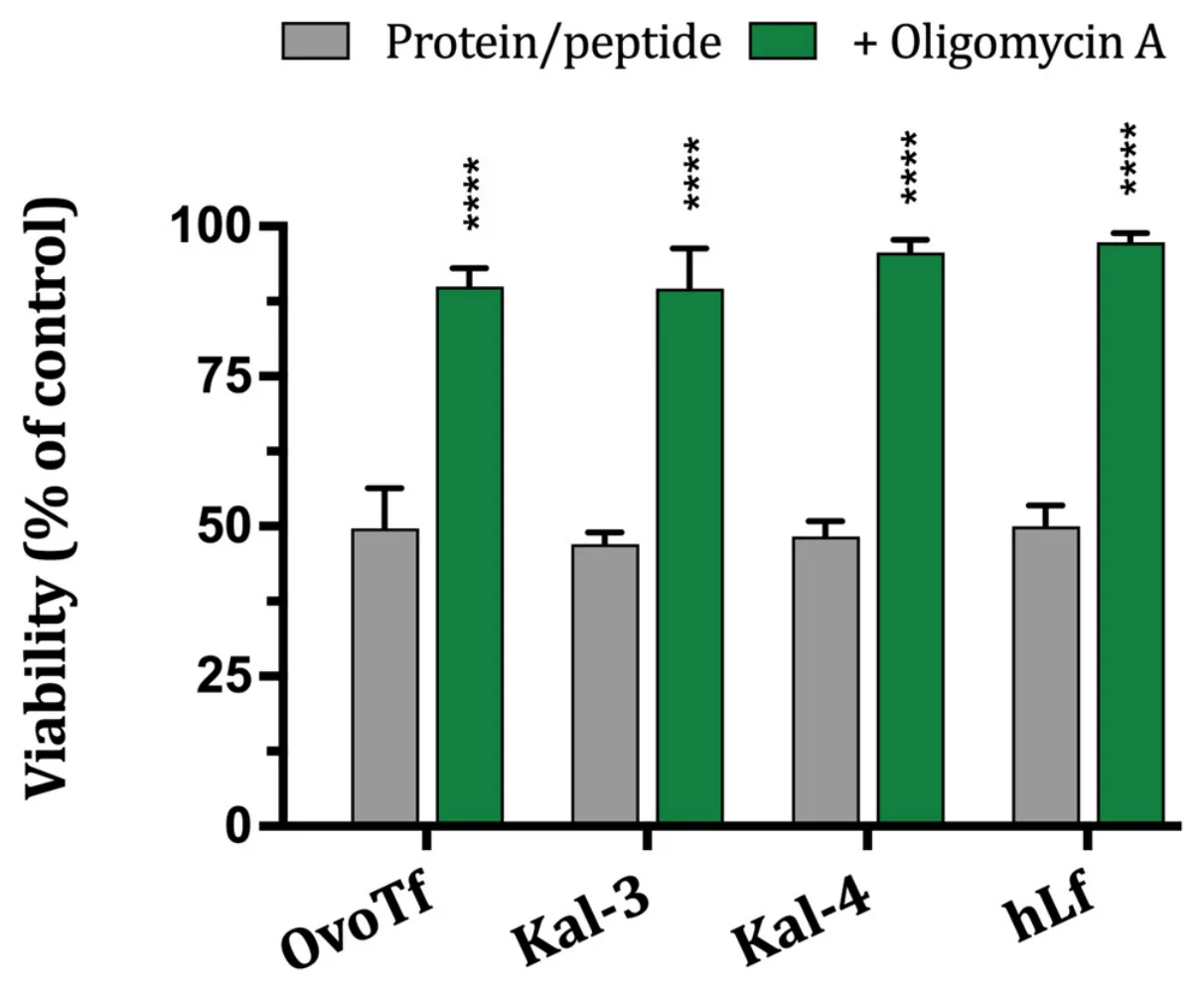
Effect of mitochondrial ATP synthase inhibition on ovotransferrin- and peptide-induced killing in *Candida albicans*. Cells were preincubated for 15 min with oligomycin A (16 μg/mL), an inhibitor of mitochondrial F_1_F_0_-ATP synthase, before exposure to ovotransferrin (OvoTf), kaliocin-3 (Kal-3), kaliocin-4 (Kal-4), or human lactoferrin (hLf) at their respective IC_50_ concentrations. Viability was determined by CFU counting and expressed relative to untreated cells. Gray bars indicate treatment with each protein or peptide alone, whereas green bars indicate the corresponding treatment after preincubation with oligomycin A. Data are presented as mean ± SD from three independent experiments. Statistical comparisons were performed using two-way ANOVA followed by Dunnett’s multiple-comparisons test, comparing each treatment in the presence of oligomycin A with the corresponding treatment alone; **** *p* < 0.0001.

Figure 5 shows that, in the absence of oligomycin A, the absence of oligomycin A, cell viability was reduced to 49.7 ± 7.0% by OvoTf, 47.0 ± 2.0% by Kal-3, and 48.3 ± 2.5% by Kal-4. Human lactoferrin, included as a reference protein, reduced viability to 50.0 ± 3.5%. These values were consistent with the use of the independently determined IC_50_ concentration of each agent. Preincubation with oligomycin A markedly attenuated these effects. Cell viability increased to 90.0 ± 3.0% in OvoTf-treated cells, 89.7 ± 6.7% in Kal-3-treated cells, and 95.7 ± 2.1% in Kal-4-treated cells (Figure 5). A similar protective effect was observed with hLf, for which viability increased to 97.3 ± 1.5%. Thus, oligomycin A increased viability by approximately 40–47 percentage points across all treatments. The protective effect was statistically significant for all four antimicrobial agents (adjusted *p* < 0.0001).

These results indicate that the candidacidal effects of OvoTf, Kal-3, and Kal-4 are modulated, at least in part, by the mitochondrial energetic state of the fungal cell. However, the protection afforded by oligomycin A does not by itself demonstrate that mitochondrial ATP synthase is a direct molecular target of these agents. Rather, mitochondrial activity may contribute indirectly to the downstream energetic and ion-homeostatic disturbances associated with their antifungal action.

### 2.5. Ovotransferrin Disrupts Pma1-Dependent Proton Homeostasis in C. albicans

The plasma membrane H^+^-ATPase Pma1 is a major ATP-consuming proton pump that drives proton extrusion across the fungal plasma membrane, thereby generating the proton-motive force, contributing to membrane potential, and regulating intracellular pH. Pharmacological impairment of *C. albicans* Pma1 has been associated with a cellular phenotype characterized by plasma membrane depolarization, intracellular ATP accumulation, and defective glucose-induced extracellular acidification [37].

**Figure 6:**
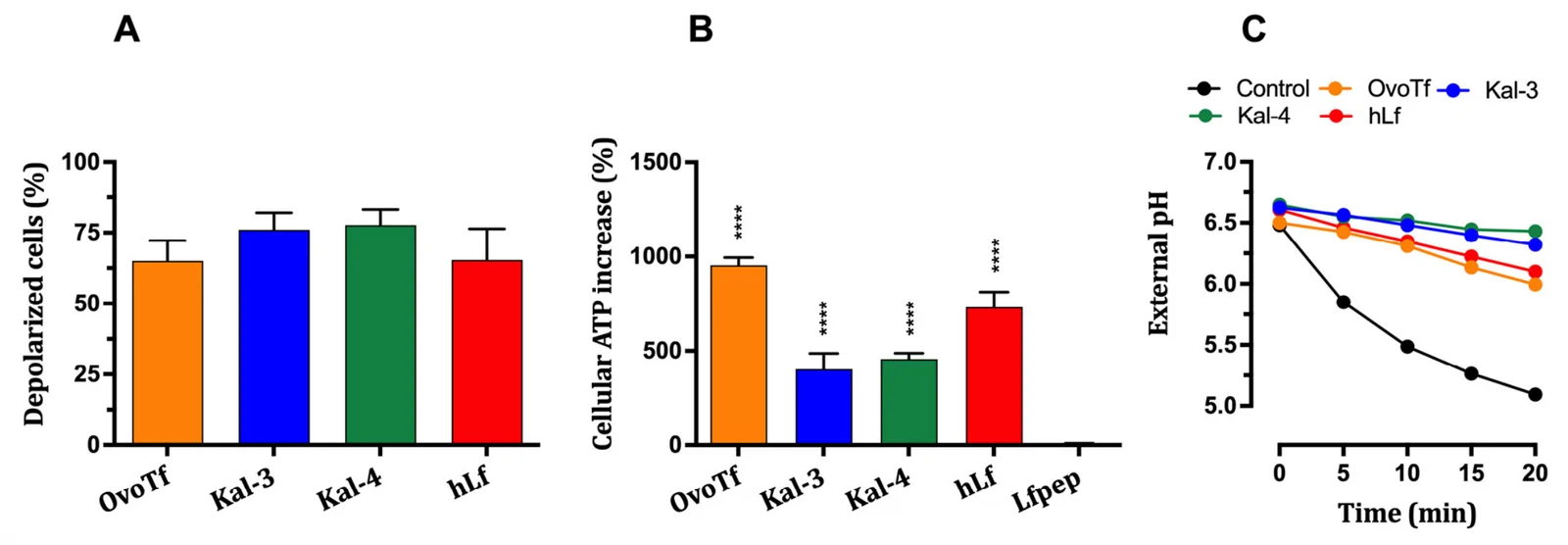
Effects of ovotransferrin and its γ-core-containing peptides on plasma membrane potential, cellular ATP accumulation, and glucose-induced extracellular acidification in *Candida albicans*. Cells were treated with ovotransferrin (OvoTf), kaliocin-3 (Kal-3), kaliocin-4 (Kal-4), human lactoferrin (hLf), or Lfpep. (**A**) Plasma membrane depolarization was assessed using DiBAC_4_(3) and expressed as the percentage of depolarized cells. (**B**) Cellular ATP content was expressed as the percentage increase relative to untreated control cells. Statistics: one-way ANOVA with Dunnett’s test versus untreated control; **** *p* < 0.0001. (**C**) Starved cells were preincubated for 15 min at 37 °C with OvoTf, Kal-3, Kal-4, or hLf at twice their respective IC_50_ concentrations. Glucose-induced extracellular acidification was monitored for 20 min after addition of glucose to a final concentration of 2.5 mM in cells suspended in 50 mM KCl. Data are presented as mean ± SD from three independent experiments. Statistical significance versus the untreated control at each time point was assessed by two-way repeated-measures ANOVA followed by Šídák’s multiple-comparisons test.

To determine whether the non-permeabilizing candidacidal activity of ovotransferrin (OvoTf) was associated with altered Pma1-dependent proton homeostasis, these functional parameters were evaluated in *C. albicans* after treatment with OvoTf, Kal-3, Kal-4, or human lactoferrin (hLf) at their respective IC_50_ concentrations. Exposure to OvoTf increased the proportion of depolarized cells to approximately 65%, whereas Kal-3, Kal-4, and hLf increased this proportion to approximately 76%, 78%, and 65%, respectively (Figure 6A). Untreated cells were used to define the polarized control population. Under the corresponding candidacidal conditions, propidium iodide uptake remained minimal, showing that substantial depolarization occurred without substantial PI-detectable plasma membrane permeabilization.

In parallel, OvoTf increased cellular ATP content by approximately 950% relative to untreated cells, corresponding to an ATP level approximately 10.5-fold that of the control (Figure 6B). hLf produced an increase of approximately 720% (8.2-fold the control level), whereas Kal-3 and Kal-4 increased ATP content by approximately 400% and 450%, respectively (approximately 5.0- and 5.5-fold the control level). Thus, ATP accumulation was numerically greater with OvoTf and hLf than with the two γ-core-containing peptides. By contrast, the permeabilizing peptide Lfpep, included as a control for a membrane-disruptive candidacidal mechanism, produced no detectable increase in cellular ATP content.

Glucose-induced extracellular acidification was then measured in starved cells as a functional readout of proton extrusion. In untreated control cells, glucose addition rapidly stimulated proton efflux, lowering the extracellular pH from 6.51 ± 0.03 at 0 min to 5.09 ± 0.09 at 20 min, corresponding to an acidification of approximately 1.4 pH units (Figure 6C). In contrast, cells preincubated with OvoTf showed a strongly attenuated acidification response, with the extracellular pH decreasing from 6.50 ± 0.02 to 6.00 ± 0.11 during the same period. Kal-3, Kal-4, and hLf similarly limited the pH decrease to approximately 0.3, 0.2, and 0.4 units, respectively. Compared with untreated cells, all four treatments maintained significantly higher extracellular pH values from 5 min onward (*p* < 0.05 at each time point), confirming a sustained reduction in glucose-induced proton extrusion. Thus, all four treatments markedly reduced the extent of glucose-induced extracellular acidification.

The proton-extrusion assay was performed in medium containing 50 mM KCl. This is important because 50 mM KCl partially protects cells from OvoTf-mediated killing. However, even under these protective ionic conditions, OvoTf, Kal-3, Kal-4, and hLf still strongly reduced glucose-induced extracellular acidification. Thus, extracellular K^+^ may help cells survive despite the disturbance in proton homeostasis, but it does not fully restore the Pma1-dependent proton-extrusion response.

Overall, OvoTf, Kal-3, Kal-4, and hLf produced a similar cellular response: plasma-membrane depolarization, ATP accumulation, and impaired glucose-induced proton extrusion, while causing little propidium iodide uptake. This phenotype is consistent with a predominantly non-permeabilizing mechanism involving disruption of fungal proton and ion homeostasis. The reduced acidification response supports impairment of Pma1-dependent proton extrusion, although direct inhibition of Pma1 was not demonstrated.

### 2.6. Ovotransferrin-Derived γ-Core-Containing Peptides Show No Visually Detectable Hemolytic Activity and Limited Effects on Mammalian Cell Metabolic Viability

**Figure 7:**
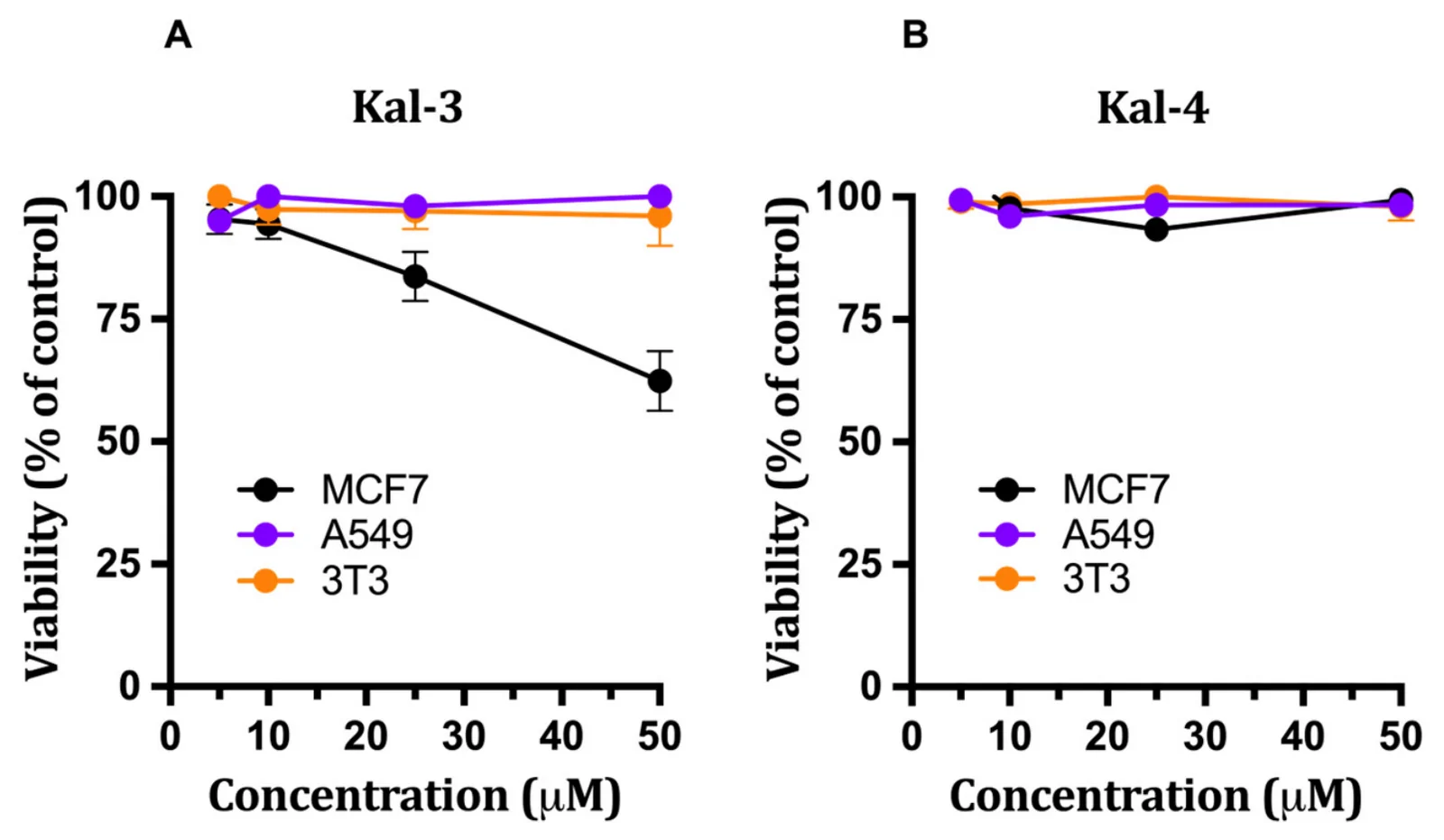
Effects of ovotransferrin-derived γ-core-containing peptides on mammalian cell metabolic viability. MCF-7, A549, and 3T3 cells were exposed to increasing concentrations of (**A**) Kal-3 or (**B**) Kal-4 for 24 h, and metabolic viability was assessed using the CCK-8 assay. Values are expressed as a percentage of the corresponding untreated control. Data are shown as mean ± SD from three independent experiments. Statistical analyses were performed by two-way ANOVA followed by Šídák’s multiple-comparisons test.

The potential toxicity of the OvoTf-derived peptides Kal-3 and Kal-4 toward mammalian cells was evaluated by assessing hemolytic activity against sheep erythrocytes and CCK-8-based metabolic viability in MCF-7, A549, and 3T3 cells. Neither peptide caused visually detectable hemolysis at concentrations up to 100 μM. After 24 h of exposure, Kal-3 produced minimal effects on metabolic viability in A549 and 3T3 cells but reduced MCF-7 viability to approximately 83% at 25 μM and 62% at 50 μM (Figure 7A). Two-way ANOVA revealed significant effects of concentration, cell line, and their interaction (all *p* < 0.0001). Šídák’s multiple-comparisons test showed that MCF-7 viability was significantly lower than that of A549 and 3T3 cells at 25 μM (*p* = 0.0004 and *p* = 0.0002, respectively) and 50 μM (both *p* < 0.0001), whereas no significant differences were observed at 5 or 10 μM. In contrast, Kal-4 did not significantly affect metabolic viability in any of the three cell lines, with no significant effects of concentration, cell line, or their interaction detected by two-way ANOVA (all *p* > 0.05; Figure 7B). Overall, both peptides exhibited no detectable hemolytic activity and limited effects on mammalian-cell metabolic viability under the conditions tested.

## 3. Discussion

### 3.1. Ovotransferrin Induces a Predominantly Non-Permeabilizing Candidacidal Phenotype

This study shows that ovotransferrin (OvoTf) exerts in vitro candidacidal activity against *Candida albicans* predominantly without PI-detectable plasma membrane permeabilization. The combined plasma membrane depolarization, ATP accumulation, impaired glucose-induced extracellular acidification, partial K^+^ release, and ionic and pharmacological protection profile points to disruption of fungal proton and ion homeostasis rather than primary lytic damage.

These results extend previous reports showing that OvoTf exerts direct antifungal activity against *Candida* spp. [7,8]. Traditionally, this activity has been linked mainly to iron sequestration. However, iron-saturated OvoTf can still retain antifungal activity, and interaction with the fungal surface has also been described [8]. Our findings provide a mechanistic explanation for this iron-independent component. They suggest that OvoTf interferes with plasma membrane-associated homeostatic functions rather than killing *C. albicans* primarily by causing large membrane lesions.

This behavior differs from the membrane-damaging activity reported for OTAP-92, a larger ovotransferrin-derived peptide active against Gram-negative bacteria [14]. These differences are not necessarily contradictory. They suggest that antimicrobial regions within ovotransferrin may act differently depending on the target organism, the structural context in which the active sequence is presented, and the physiological state of the cell.

The comparison with human lactoferrin (hLf) was especially useful. hLf was included as a predominantly non-permeabilizing reference because its candidacidal activity has previously been associated with defective Pma1-dependent proton extrusion, altered ion homeostasis, ATP accumulation, and mitochondrial involvement in cell death [27,28,29]. In contrast, Lfpep was used as a membrane-permeabilizing comparator. OvoTf behaved more like hLf than like Lfpep across all measured parameters. This does not mean that OvoTf and hLf necessarily bind to the same initial target. Rather, it suggests that both proteins converge on a similar functional outcome: disruption of fungal proton and ion homeostasis.

### 3.2. Impairment of Pma1-Dependent Proton and Ion Homeostasis

The simultaneous presence of membrane depolarization, ATP accumulation, and defective extracellular acidification is consistent with impaired Pma1-dependent proton extrusion. Pma1 is the major fungal plasma membrane H^+^-ATPase. It uses ATP to extrude protons and thereby helps maintain cytosolic pH, membrane potential, and the proton gradient required for secondary transport [32,33,34]. Therefore, a defect in Pma1-dependent proton extrusion would be expected to affect several cellular processes at the same time. In this context, ATP accumulation may reflect continued ATP production together with reduced ATP consumption by Pma1-driven proton pumping.

A similar combination of reduced proton extrusion, membrane depolarization, and ATP accumulation has been described for hLf, human defensins, some antimicrobial γ-core peptides, and BM2, a peptide previously identified as a potent inhibitor of the fungal plasma membrane H^+^-ATPase Pma1 [27,30,31,37]. This supports the idea that this phenotype is not simply a nonspecific consequence of cell death, but rather a reproducible signature of impaired proton homeostasis [37]. However, our data do not demonstrate direct enzymatic inhibition of Pma1. OvoTf and its derived peptides could affect proton extrusion by directly interacting with Pma1, by modifying its membrane environment, by altering regulatory pathways, or by a combination of these mechanisms.

An indirect mechanism involving the membrane environment is plausible. Pma1 activity depends not only on the enzyme itself but also on its organization within the fungal plasma membrane and on the lipid composition of surrounding microdomains. Changes in ergosterol-rich domains can alter Pma1 localization or function without requiring direct inhibition of its catalytic site [38,39]. OvoTf and its γ-core-containing peptides could therefore impair proton extrusion by disturbing local protein–lipid organization. This possibility is also consistent with previous observations for hLf and other host-defense effectors that alter fungal proton homeostasis without causing extensive membrane permeabilization [27,30,31,39]. The present functional assays establish impairment of Pma1-dependent proton extrusion but do not distinguish between direct catalytic inhibition and indirect disruption of the membrane organization required for normal Pma1 activity.

### 3.3. Ionic Conditions and K^+^ Transport Modulate the Lethal Response

The salt experiments further support a role for ion homeostasis. KCl and NaCl strongly reduced the candidacidal activity of OvoTf, Kal-3, and Kal-4. In contrast, sorbitol adjusted to comparable osmolality did not provide similar protection. Thus, protection cannot be explained simply by non-ionic osmotic stabilization. Increased ionic strength may reduce electrostatic interactions between the antifungal agents and the fungal surface, especially for positively charged regions such as Kal-3. However, this explanation is unlikely to fully account for the protective effect of salts. First, protection was also observed with the nearly neutral Kal-4. Second, glucose-induced extracellular acidification was impaired in medium containing 50 mM KCl, a concentration that attenuated OvoTf-mediated killing. Therefore, protective KCl concentrations do not prevent OvoTf and its γ-core-containing peptides from functionally affecting the Pma1-dependent proton-extrusion response. This argues against a simple model in which increased ionic strength protects cells only by preventing interaction of these molecules with the fungal surface or plasma membrane. Rather, extracellular cations may attenuate killing by buffering downstream ionic imbalance or by reducing the lethal consequences of impaired proton homeostasis. The effect of TEA further suggests that ion-dependent cellular responses, including K^+^ efflux pathways, also contribute. Similar salt protection, but not sorbitol protection, has been reported for hLf and human defensins [27,30], further linking OvoTf activity to a broader mechanism involving fungal ion homeostasis.

OvoTf also induced extracellular K^+^ release corresponding to approximately 24% of the total cell-associated K^+^ pool. This partial release may reflect limited membrane leakage below the threshold required for PI entry, activation of endogenous K^+^ transport pathways, or both. TEA strongly protected cells against OvoTf-, Kal-3-, and Kal-4-mediated killing, supporting a contribution of TEA-sensitive K^+^ transport processes or K^+^-dependent homeostatic responses. This agrees with previous studies showing that TEA attenuates killing by hLf, human defensins, and other γ-core-containing antimicrobial peptides [30,31,40]. Previous work has also linked the fungal K^+^ channel Tok1 to susceptibility to hLf and histatin 5 [40,41]. Together, these observations suggest that K^+^ loss, or the cellular response to K^+^ imbalance, may amplify the activity of several non-permeabilizing antifungal effectors.

The glucose-induced extracellular acidification assay further separated impaired proton extrusion from irreversible loss of viability. OvoTf, Kal-3, Kal-4, and hLf markedly reduced acidification even in the presence of 50 mM KCl, a condition that partially protected cells from killing in the salt screen. Thus, extracellular K^+^ did not fully prevent these agents from affecting the Pma1-associated acidification response. Rather, K^+^ may reduce the downstream ionic imbalance that converts defective proton extrusion into lethal damage, as previously proposed for hLf and γ-core-containing peptides [27,30,31]. This interpretation is consistent with the Nernst relationship for K^+^, whereby changes in extracellular K^+^ modify the electrochemical gradient for K^+^ movement [42]. Accordingly, extracellular K^+^ does not need to approach intracellular concentrations to be protective; even moderate supplementation may reduce the driving force for K^+^ efflux and limit net K^+^ loss during OvoTf-induced ionic stress. In this framework, impaired Pma1-dependent proton extrusion and irreversible cell death are linked but separable steps: extracellular ions may buffer the consequences of defective proton pumping without fully restoring the proton-extrusion response.

### 3.4. Mitochondrial Bioenergetic Activity Is Required for, but Is Not the Primary Target of Candidacidal Killing

The mitochondrial experiments indicate that mitochondrial bioenergetic activity is functionally required for the full candidacidal response under the conditions tested, although mitochondria do not appear to be the primary molecular target of OvoTf. Piericidin A and oligomycin A markedly increased survival after exposure to OvoTf, Kal-3, or Kal-4. However, none of these agents measurably reduced oxygen consumption, whereas piericidin A produced the expected inhibition of respiration. These findings argue against direct suppression of the electron transport chain as the main antifungal mechanism. Instead, respiratory and F_1_F_0_-ATP synthase activities appear to constitute a critical downstream step determining whether the plasma membrane-associated homeostatic disturbance progresses to irreversible cell death.

At first sight, protection by respiratory or ATP synthase inhibition may seem paradoxical, because metabolically active cells are usually expected to tolerate stress better. One working model is that, under the controlled assay conditions used here, defective Pma1-dependent proton extrusion turns ongoing metabolic activity into an amplifier of homeostatic stress rather than a corrective response. Continued ATP generation, respiratory activity, and ion movements could increase the burden on cytosolic and mitochondrial pH/ion regulation while plasma membrane proton export remains impaired. In this context, the cell may eventually cross a threshold beyond which the disturbance becomes irreversible. By slowing these fluxes, piericidin A or oligomycin A may delay or reduce the likelihood of reaching this threshold.

Within this framework, the ATP accumulation described above (Section 3.2) is compatible with continued respiratory activity maintaining the mitochondrial proton-motive force and favoring proton influx into the matrix through F_1_F_0_-ATP synthase. If plasma membrane proton extrusion remains impaired and ionic compensation is insufficient, sustained ATP synthase-dependent proton influx could overwhelm mitochondrial pH homeostasis and constitute the critical downstream event that drives the cell beyond the lethal homeostatic threshold. The strong protection afforded by piericidin A and oligomycin A therefore suggests that mitochondrial respiratory and ATP synthase activities are functionally required for the full candidacidal response under the conditions tested. However, because mitochondrial pH was not directly measured, matrix acidification remains a mechanistic hypothesis rather than a demonstrated event. Thus, mitochondria are not proposed as the primary molecular target of OvoTf, but as a critical downstream amplifier and execution component of the lethal response. Comparable ATP accumulation and mitochondrial participation have been reported in *C. albicans* exposed to hLf, defensins, the BM2 peptide, and pharmacological inhibitors of Pma1 [27,28,30,31,37].

**Figure 8:**
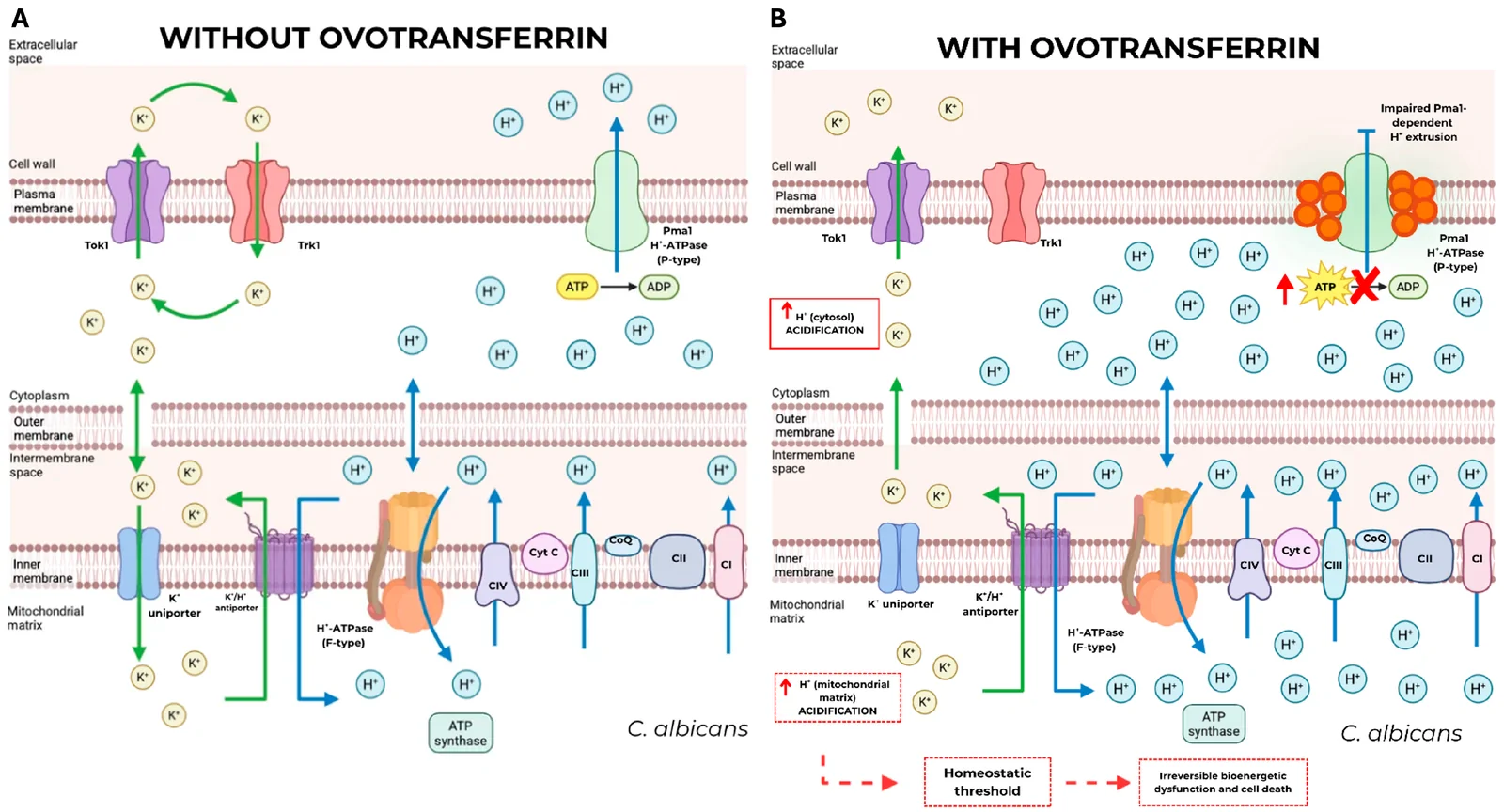
Proposed homeostatic-threshold model linking ovotransferrin-induced K^+^/H^+^ imbalance to mitochondrial pH dysregulation and cell death in *Candida albicans*. (**A**) In untreated cells, the plasma-membrane H^+^-ATPase Pma1 extrudes cytosolic protons and contributes to intracellular pH regulation, membrane potential, and the proton gradient required for secondary transport. This Pma1-dependent electrochemical balance is functionally linked to K^+^ homeostasis through K^+^ uptake and efflux pathways. In mitochondria, respiratory-chain complexes pump protons from the matrix to the intermembrane space, whereas F_1_F_0_-ATP synthase uses proton re-entry into the matrix to synthesize ATP. Mitochondrial K^+^ uptake and K^+^/H^+^ exchange are shown as generic processes contributing to matrix ion balance, volume regulation, and mitochondrial membrane potential. (**B**) After ovotransferrin exposure, cells show impaired glucose-induced proton extrusion, plasma-membrane depolarization, ATP accumulation, and partial extracellular K^+^ release. These effects are represented as disruption of Pma1-dependent proton homeostasis and altered K^+^ handling, without implying direct inhibition of Pma1 or involvement of a specific K^+^ transport protein. ATP accumulation is consistent with continued ATP synthesis and reduced Pma1-dependent ATP consumption. The protective effects of TEA, piericidin A, and oligomycin A are represented as evidence that K^+^ transport processes and mitochondrial bioenergetic activity are functionally required for the full lethal response under the conditions tested. The model proposes that, when plasma membrane proton extrusion remains impaired and ionic compensation is insufficient, continued proton entry into the mitochondrial matrix through F_1_F_0_-ATP synthase may overwhelm mitochondrial pH regulation and drive the cell beyond the lethal homeostatic threshold. This mitochondrial event is proposed as a mechanistic hypothesis and was not directly demonstrated in the present study. Upward arrows indicate an increase in the corresponding parameter; the red cross denotes impaired Pma1-dependent proton extrusion. Dashed red arrows indicate the proposed progression toward the lethal homeostatic threshold.

The homeostatic-threshold model summarized in Figure 8 integrates these observations into a single framework. Experimentally, OvoTf treatment is associated with impaired glucose-induced proton extrusion, plasma membrane depolarization, ATP accumulation, partial extracellular K^+^ release, protection by salts and TEA, and reduced susceptibility after inhibition of respiration or ATP synthase. Together, these findings support a model in which the initial disturbance is centered on plasma membrane proton homeostasis, whereas K^+^ redistribution and mitochondrial bioenergetic activity govern its progression from a potentially reversible adaptive imbalance to irreversible cell death. In this framework, continued proton entry into the mitochondrial matrix through F_1_F_0_-ATP synthase is proposed as a critical downstream event that may overwhelm mitochondrial pH regulation once cellular compensatory capacity is exceeded. Because mitochondrial pH was not directly measured, this specific step remains a mechanistic hypothesis to be tested.

### 3.5. Structural and Functional Implications of Kal-3 and Kal-4

The identification of Kal-3 and Kal-4 adds a structural dimension to this mechanism. Kal-3 is located within the previously described OTAP-92 antimicrobial region, whereas Kal-4 is a distinct γ-core-containing sequence in the C-lobe of OvoTf. The γ-core motif has been proposed as a conserved structural signature associated with antimicrobial activity in many cysteine-rich peptides and proteins [18]. Kal-3 lies within OTAP-92, a cysteine-rich 92-residue N-lobe fragment containing three intrachain disulfide bonds, whose reduction abolished its antibacterial activity [13]. OTAP-92 also contains helix–sheet elements resembling those of insect defensins and disrupts bacterial membranes [14]. The identification of candidacidal activity within the shorter Kal-3 region suggests that its γ-core-containing segment may contribute to the antimicrobial potential of the broader OTAP-92 domain.

The activity of both Kal-3 and Kal-4 is relevant because these peptides are located in different lobes of OvoTf but share a γ-core-containing organization. Their candidacidal activity reinforces the association between the γ-core motif and antimicrobial function in distinct regions of host-defense proteins. However, this should not be interpreted to mean that the γ-core motif alone is sufficient for activity, or that all γ-core-containing sequences act through the same mechanism. More likely, the γ-core provides a structural scaffold, while the surrounding sequence, physicochemical properties, and structural context determine the final antimicrobial phenotype [18,19,20,21,22,23,24,31].

Kal-3 and Kal-4 reproduced several key features of full-length OvoTf, including concentration-dependent candidacidal activity, limited PI uptake, plasma membrane depolarization, impaired extracellular acidification, and similar modulation by ionic and bioenergetic conditions. However, ATP accumulation induced by these peptides was lower than that induced by full-length OvoTf and hLf. This difference indicates that the peptides do not fully reproduce the phenotype of the parental protein.

These findings support the idea that large host-defense proteins can contain embedded antimicrobial modules that retain selected properties of the parental molecule. Ovotransferrin may therefore act both as a full-length antifungal effector and as a source of smaller bioactive sequences. The absence of visually detectable hemolysis and the limited effects on mammalian cell viability provide preliminary support for further evaluation of Kal-3 and Kal-4 as non-lytic antifungal templates. However, these results do not establish therapeutic safety or in vivo efficacy.

### 3.6. Study Scope, Limitations, and Future Directions

The present study was designed as a mechanistic analysis using the *C. albicans* reference strain ATCC 10231. Further studies including clinical isolates and other *Candida* species will help determine the broader applicability of the observed phenotype, while genetic and biochemical approaches will be required to identify the specific molecular components involved. Overall, these findings place OvoTf among a broader group of host-defense effectors, including hLf and human defensins, whose in vitro candidacidal activity has been shown to occur without PI-detectable plasma membrane permeabilization and to be linked to disruption of fungal proton and ion homeostasis.

The proposed homeostatic-threshold model provides a testable framework to explain how an initially non-lytic disturbance of Pma1-dependent proton extrusion and K^+^ balance can become lethal when mitochondrial bioenergetic activity and associated proton and ion fluxes drive the imbalance beyond the compensatory capacity of the cell. Within this framework, Kal-3 and Kal-4 support the γ-core motif as a conserved structural scaffold whose antimicrobial activity depends on sequence context, physicochemical properties, and structural presentation. Thus, OvoTf emerges not only as an antifungal protein but also as a reservoir of structurally encoded effector modules that exploit a conserved vulnerability of fungal pH and ion regulation. This view may help guide the design of new non-lytic antifungal strategies based on controlled disruption of fungal homeostatic networks.

## 4. Materials and Methods

### 4.1. Materials

Apo-ovotransferrin from chicken egg white (OvoTf; UniProt accession number P02789) and human apo-lactoferrin (hLf; UniProt accession number P02788) were purchased from Merck KGaA (Darmstadt, Germany). The ovotransferrin-derived peptides kaliocin-3 (Kal-3; residues 155–183 of mature ovotransferrin, corresponding to UniProt P02789 residues 174–202) and kaliocin-4 (Kal-4; residues 484–514 of mature ovotransferrin, corresponding to UniProt P02789 residues 503–533), together with Lfpep, a membrane-permeabilizing peptide corresponding to residues 18–40 of mature human lactoferrin (UniProt P02788 residues 37–59; sequence TKCFQWQRNMRKVRGPPVSCIKR), were synthesized by GenScript Biotech Corp. (Piscataway, NJ, USA) at ≥95% purity, as determined by reverse-phase high-performance liquid chromatography (RP-HPLC). Lfpep was included as a positive control for membrane-permeabilizing candidacidal activity [43,44].

Oligomycin A, piericidin A, tris(hydroxymethyl)aminomethane (Tris), tetraethylammonium chloride (TEA), glucose, sorbitol, and Triton X-100 were purchased from Sigma-Aldrich/Merck KGaA (Darmstadt, Germany). DiBAC_4_(3) and propidium iodide (PI) were obtained from Thermo Fisher Scientific–Molecular Probes (Eugene, OR, USA). The BacTiter-Glo™ Microbial Cell Viability Assay was obtained from Promega (Madison, WI, USA). Sabouraud dextrose broth (SDB) and Sabouraud dextrose agar (SDA) were purchased from Oxoid (Basingstoke, UK). Defibrinated sheep blood (SR0051C; Thermo Scientific Oxoid) was obtained from Fisher Scientific Spain (Madrid, Spain), and the Cell Counting Kit-8 (CCK-8) was purchased from Sigma-Aldrich/Merck KGaA. All other reagents were of analytical grade.

Peptides were dissolved in sterile ultrapure water to prepare stock solutions, divided into single-use aliquots, and stored at −20 °C until use. Piericidin A and oligomycin A stock solutions were prepared in ethanol. Vehicle-matched controls containing the same final concentration of the corresponding solvent were included in all experiments.

### 4.2. Candida albicans Strain and Growth Conditions

*Candida albicans* ATCC 10231 was obtained from the American Type Culture Collection (ATCC) and maintained as frozen glycerol stocks. Cells were routinely recovered on SDA plates at 30 °C. For experimental assays, cells were grown aerobically in SDB at 30 °C with orbital shaking at 150 rpm for 16–20 h. An aliquot of the overnight culture was subsequently transferred to fresh SDB and grown under the same conditions until the mid-exponential phase.

### 4.3. In Silico Sequence, Physicochemical, and Structural Analyses

The chicken (*Gallus gallus*) ovotransferrin amino acid sequence was retrieved from UniProt (UniProt accession P02789). Because UniProt P02789 includes a 19-residue signal peptide, residue numbering for OTAP-92, Kal-3, and Kal-4 is reported according to the mature ovotransferrin chain, unless otherwise indicated. Thus, mature residue 1 corresponds to UniProt residue 20.

Kal-3 and Kal-4 correspond to γ-core-containing regions of ovotransferrin previously identified according to the sequence–structure criteria described by Yount and Yeaman [18] and subsequently applied to transferrin-family proteins in our earlier study [17]. In the present work, these regions were mapped onto the experimentally determined OvoTf structure to define their location within the N- and C-terminal lobes and examine their presentation in the native protein. For comparative structural analysis, the previously described γ-core-containing regions of human lactoferrin were mapped onto the experimentally determined structure of human apolactoferrin (PDB ID: 1CB6) [45]. OvoTf and human lactoferrin were represented using the same orientation, structural-display settings, and color-coding criteria to facilitate direct comparison of the location and surface presentation of the corresponding regions.

Structural representations of ovotransferrin and the mapped OTAP-92, Kal-3, and Kal-4 regions were generated in UCSF ChimeraX version 1.10 (University of California, San Francisco, CA, USA) using the experimentally determined X-ray crystal structure of chicken apo-ovotransferrin (PDB ID: 1AIV) [46]. Peptide regions were mapped onto the protein structure after conversion between mature-chain and UniProt precursor residue numbering, as required.

Multiple sequence alignments were generated with Clustal Omega through the EMBL-EBI Job Dispatcher (https://www.ebi.ac.uk/jdispatcher/msa/clustalo; accessed on 23 December 2025) using default parameters. The resulting alignment was visualized and formatted in Jalview version 2.11.5.1 (University of Dundee, Dundee, UK) to highlight the correspondence between Kal-3 and its parent OTAP-92 region and to improve graphical clarity.

Physicochemical parameters, including peptide length, average molecular mass (*m*_av_), net charge at pH 7.0, and relative hydrophobicity (*H*_R_), were calculated from the primary amino acid sequence of each peptide using the Prot pi Peptide Tool (Prot pi, Zurich, Switzerland; https://www.protpi.ch) and the Biosynth Peptide Calculator (Biosynth, Staad, Switzerland; https://www.biosynth.com/peptide-calculator), both accessed on 20 April 2026.

Helical-wheel projections of Kal-3 and Kal-4 were generated using NetWheels online tool (University of Brasília, Brasília, Brazil; http://lbqp.unb.br/NetWheels, accessed on 29 January 2026). The projections were used to visualize the spatial distribution of basic, acidic, polar uncharged, and nonpolar residues under an idealized α-helical model. They were interpreted solely as theoretical representations of potential amphipathic residue segregation and not as evidence that the isolated peptides adopt an α-helical conformation.

### 4.4. Candidacidal Assays

Susceptibility to ovotransferrin and the derived peptides was assessed using a colony-counting assay, as previously described with minor modifications [27]. Briefly, *C. albicans* suspensions were adjusted to an estimated density of 1 × 10^6^ cells/mL based on optical density measurements at 620 nm and resuspended in 10 mM Tris–HCl buffer, pH 7.4 (Tris buffer). Cells were incubated with the indicated concentrations of the tested proteins or peptides for 90 min at 37 °C. Following treatment, aliquots were serially diluted, and 100 μL of the appropriate dilutions were plated in duplicate on SDA plates. Plates were incubated for 24–48 h at 30 °C, and plates containing 50–200 colonies were used for enumeration. Viable cell counts were determined by colony-forming unit (CFU) enumeration and expressed as the percentage of survival relative to the untreated control. Three independent experiments were performed, with each dilution plated in duplicate, and the results are presented as mean ± standard deviation (SD). Vehicle-matched controls were included in all assays involving inhibitors prepared in organic solvent.

Dose–response curves were fitted by nonlinear regression using a four-parameter logistic model with variable slope in GraphPad Prism version 11.0.2 (GraphPad Software, San Diego, CA, USA). Half-maximal inhibitory concentrations (IC_50_) were estimated from the fitted curves using survival data expressed relative to the corresponding untreated controls.

For pharmacological modulation assays, unless otherwise specified, cell suspensions were preincubated for 15 min at 37 °C with oligomycin A (16 μg/mL), piericidin A (16 μM), or tetraethylammonium chloride (TEA; 10 mM) before addition of the antimicrobial protein or peptide. Parallel controls containing each pharmacological agent in the absence of the antimicrobial treatment were included. Human lactoferrin (hLf) and Lfpep were used as reference compounds for predominantly non-permeabilizing and membrane-permeabilizing candidacidal mechanisms, respectively.

To distinguish ionic effects from those caused by increased osmolality, KCl and NaCl were initially screened at concentrations ranging from 0 to 150 mM, in 10 mM increments. Subsequent comparative candidacidal assays were performed in Tris buffer supplemented with 150 mM KCl or 140 mM NaCl. In parallel, sorbitol adjusted to 300 mOsm kg^−1^ H_2_O was used as a non-ionic osmolality control. For assays containing salts, sorbitol, or TEA, viability was expressed relative to the corresponding control containing the same additive but no antimicrobial protein or peptide. Osmolality was measured in triplicate using an automatic osmometer (OM-6060; Arkray Inc., Kyoto, Japan) calibrated with purified water and commercial standard solutions.

### 4.5. Plasma Membrane Permeabilization Assay

Plasma membrane permeabilization was evaluated by flow-cytometric analysis of PI uptake. *C. albicans* cells (10^6^ cells/mL) suspended in Tris buffer, were incubated with the indicated concentrations of OvoTf, Kal-3, Kal-4, hLf, or Lfpep for 90 min at 37 °C. PI was then added to a final concentration of 1 μg/mL, and samples were incubated for 5 min in the dark. Fluorescence was measured using a CytoFLEX S flow cytometer (Beckman Coulter Life Sciences, Indianapolis, IN, USA), with excitation at 638 nm and emission collected through a 780/60 nm band-pass filter. A total of 5000 cellular events were acquired for each sample. Forward- and side-scatter parameters were used to exclude debris and cell aggregates. Data were collected and analyzed using CytExpert version 2.1 software (Beckman Coulter Life Sciences, Indianapolis, IN, USA). Unstained cells were used to establish background fluorescence and define the PI-positive population. Results were expressed as the percentage of PI-positive cells. Experiments were performed independently three times, and data are presented as mean ± SD. Lfpep was included as a membrane-permeabilizing positive control, whereas untreated and unstained cells were used to establish background fluorescence and the PI-positive threshold.

### 4.6. Measurement of Extracellular Potassium Release

Extracellular K^+^ release was measured according to a previously reported procedure with minor modifications [41]. Briefly, *C. albicans* cells were grown in Sabouraud dextrose broth (SDB) for 16–20 h at 30 °C and subsequently subcultured in fresh SDB until the mid-exponential phase. Cells were harvested, washed twice with sterile ultrapure water, and adjusted to a final density of 1 × 10^7^ cells/mL. Cell suspensions were incubated with OvoTf at its 2 × IC_50_ concentration for 90 min at 37 °C. The samples were then centrifuged at 1100× *g* for 10 min, and the cell-free supernatants were collected and stored at 4 °C until analysis. Extracellular K^+^ was quantified by inductively coupled plasma–optical emission spectrometry (ICP–OES) using an Optima 2000 DV instrument (PerkinElmer, Waltham, MA, USA), with yttrium as an internal standard.

Total cell-associated K^+^, defined as 100%, was determined in parallel untreated cell suspensions treated with 0.5% (*v*/*v*) perchloric acid for 1 h at 95 °C. Samples were then centrifuged to remove cellular debris, and K^+^ was quantified in the resulting supernatants. Extracellular K^+^ release was calculated as the amount of K^+^ detected in the extracellular supernatant expressed as a percentage of the total cell-associated K^+^ content measured in the corresponding cell suspension. Reagent and buffer blanks were subtracted before calculation of the percentage of released K^+^. Data are presented as mean percentage K^+^ release ± SD from three independent experiments, each performed in duplicate.

### 4.7. Plasma Membrane Potential Assay

Changes in the plasma membrane potential of *C. albicans* were assessed using the anionic voltage-sensitive fluorescent probe DiBAC_4_(3), following a previously reported protocol [30] with minor modifications. Briefly, mid-exponential-phase cells were collected by centrifugation, washed twice, and resuspended in Tris buffer at a final density of 10^6^ cells/mL. Cell suspensions were incubated for 90 min at 37 °C with ovotransferrin (OvoTf), kaliocin-3 (Kal-3), kaliocin-4 (Kal-4), or human lactoferrin (hLf) at their respective IC_50_ concentrations. DiBAC_4_(3) was then added to a final concentration of 1 µg/mL, and samples were incubated for 30 min at 37 °C in the dark.

Fluorescence was quantified by flow cytometry using a CytoFLEX S instrument (Beckman Coulter Life Sciences, Indianapolis, IN, USA), with excitation at 561 nm and emission collected through a 690/50 nm band-pass filter. A total of 5000 cellular events were acquired for each sample. Forward- and side-scatter parameters were used to exclude debris and cell aggregates. Increased DiBAC_4_(3) fluorescence was interpreted as plasma membrane depolarization. Untreated cells were used to establish the polarized reference population and to define the fluorescence threshold for classification of depolarized cells. Data acquisition and analysis were performed using CytExpert version 2.1 software. Human lactoferrin was included as a non-permeabilizing positive reference for plasma membrane depolarization. Experiments were performed independently three times, and results are expressed as the mean percentage of depolarized cells ± SD.

### 4.8. Oxygen Consumption Measurements

Oxygen consumption was monitored as previously described [31], with minor modifications, using a biological oxygen monitor (Digital Model 20, Rank Brothers Ltd., Cambridge, UK) equipped with a closed, temperature-controlled dual-chamber system that enabled simultaneous recording of treated and untreated samples. Cells grown to mid-exponential phase in SDB were harvested, washed twice with Tris buffer, and resuspended in the same buffer at a final density of 5 × 10^6^ cells/mL. Cell suspensions were preincubated for 15 min at 37 °C with ovotransferrin (OvoTf), kaliocin-3 (Kal-3), or kaliocin-4 (Kal-4), each used at twice its respective IC_50_ concentration, or with piericidin A (PierA; 32 µM). These concentrations had previously been confirmed not to cause substantial PI uptake at the cell density used. Oxygen consumption was recorded at 25 °C at 5 min intervals for 15 min and expressed as the percentage of oxygen saturation over time. Oxygen saturation values at each time point are presented as mean ± SD from three independent experiments.

### 4.9. Measurement of Cellular ATP Levels

Cellular ATP content was quantified as previously described [31], with minor modifications, using the BacTiter-Glo™ Microbial Cell Viability Assay (Promega) according to the manufacturer’s instructions. Briefly, *C. albicans* cells were grown to mid-exponential phase in SDB at 30 °C, washed twice with Tris buffer, pH 7.4, and resuspended at a final density of 10^6^ cells/mL. Aliquots were incubated for 90 min at 37 °C with ovotransferrin (OvoTf), kaliocin-3 (Kal-3), kaliocin-4 (Kal-4), human lactoferrin (hLf), or the membrane-permeabilizing peptide Lfpep, each at its independently determined IC_50_ value. Samples (100 μL) were transferred to opaque white 96-well plates, mixed with an equal volume of BacTiter-Glo™ reagent, and incubated for 15 min in the dark. Luminescence was measured using a Varioskan Flash multimode reader (Thermo Scientific, Waltham, MA, USA) after 10 s of shaking, with an integration time of 1 s. Calibration curves were generated in each experiment using ATP standards ranging from 1 to 1000 nM. Cellular ATP concentrations were interpolated from the corresponding standard curves and expressed as the percentage increase relative to untreated control cells, calculated as [(ATP-treated − ATP-control)/ATP-control] × 100. Three independent experiments were performed, each with duplicate determinations.

### 4.10. Measurement of Glucose-Induced Extracellular Acidification

To assess Pma1-dependent proton extrusion, glucose-induced extracellular acidification was measured as previously described by Martínez-Muñoz and Kane, with minor modifications [32]. Briefly, mid-exponential-phase *C. albicans* cells were washed twice with 10 mM Tris–HCl buffer, pH 8.0, and the optical density of the cell suspension was adjusted to 0.5 at 620 nm. Cells were then resuspended in 50 mM KCl and incubated for 18 h at 4 °C under starvation conditions to reduce basal metabolic activity.

For extracellular acidification measurements, starved cells were adjusted to approximately 1 × 10^7^ cells/mL and incubated for 15 min at 37 °C in the presence or absence of ovotransferrin (OvoTf), kaliocin-3 (Kal-3), kaliocin-4 (Kal-4), or human lactoferrin (hLf), each at twice its independently determined IC_50_ concentration. Human lactoferrin was included as a predominantly non-permeabilizing reference comparator. The initial pH of each suspension was adjusted to approximately 6.6 with HCl, after which glucose was added to a final concentration of 2.5 mM. Extracellular pH was recorded every 5 min for 20 min using a SevenMulti S50-K pH meter (Mettler-Toledo, Greifensee, Switzerland) equipped with a calibrated electrode, under continuous stirring. Three independent experiments were performed.

### 4.11. Hemolysis Assay

The hemolytic activity of the ovotransferrin-derived peptides kaliocin-3 (Kal-3) and kaliocin-4 (Kal-4) was evaluated qualitatively using defibrinated sheep blood, based on previously reported sheep erythrocyte assays [47]. Defibrinated sheep blood (SR0051C; Thermo Scientific Oxoid) was centrifuged at 1000× *g* for 15 min at 4 °C, and the erythrocyte pellet was washed once with phosphate-buffered saline (PBS; pH 7.4) and recentrifuged under the same conditions. The final erythrocyte pellet was resuspended in PBS. Twofold serial dilutions of Kal-3 and Kal-4, with a maximum concentration of 100 μM, were prepared in 96-well plates. PBS and Triton X-100 (0.1%) were used as negative and positive hemolysis controls, respectively. An aliquot (100 μL) of the erythrocyte suspension was then added to each well. Plates were incubated for 30 min at 37 °C and subsequently maintained at room temperature for 24 h. Hemolysis was evaluated visually based on the presence of a compact erythrocyte pellet and the absence of visible hemoglobin release into the supernatant. The assay was therefore interpreted qualitatively as detectable or non-detectable hemolysis. Three independent experiments were performed.

### 4.12. Cytotoxicity Assay

The mammalian-cell metabolic-viability assays were performed at the Unit of Biotechnology and Biomedicine, Scientific-Technical Services, University of Oviedo (SCT-UNIOVI, Oviedo, Spain). The effects of the ovotransferrin-derived peptides kaliocin-3 (Kal-3) and kaliocin-4 (Kal-4) were evaluated using the human breast adenocarcinoma cell line MCF-7, the human lung adenocarcinoma cell line A549, and mouse 3T3 fibroblasts, included as a non-tumor fibroblast model. These cell-lines were obtained from ATCC (Manassas, VA, USA). Cells were maintained in Dulbecco’s modified Eagle’s medium (DMEM) supplemented with 10% fetal bovine serum (FBS) at 37 °C in a humidified atmosphere containing 5% CO_2_.

Cells were seeded in 96-well plates at a density of 5 × 10^2^–1 × 10^3^ cells per well and allowed to adhere for 24 h. Kal-3 and Kal-4 were dissolved in phosphate-buffered saline (PBS) and added at final concentrations of 5, 10, 25, and 50 μM. Control wells received an equivalent volume of PBS without peptide. After 24 h of peptide exposure, metabolic viability was assessed using the Cell Counting Kit-8 (CCK-8) according to the manufacturer’s instructions. After incubation with the CCK-8 reagent, absorbance was measured at 450 nm using a SynergyLX microplate reader (BioTek, Agilent Technologies, Santa Clara, CA, USA). Results were expressed as a percentage of the corresponding untreated control. Three independent experiments were performed, with three technical replicates per condition.

### 4.13. Statistical Analysis

Data were analyzed using GraphPad Prism version 11.0.2 (GraphPad Software, San Diego, CA, USA). Technical replicates were averaged to obtain a single value for each independent experiment and were not treated as independent observations. Unless otherwise indicated, data are presented as mean ± SD from three independent experiments. Dose–response curves were fitted by nonlinear regression using a four-parameter logistic model to estimate IC_50_ values. Group comparisons were performed using one-way or two-way ANOVA, followed by Dunnett’s or Šídák’s multiple-comparisons test, as indicated in the corresponding figure legends. Time-course experiments were analyzed by two-way repeated-measures ANOVA followed by the multiple-comparisons test specified in the corresponding figure legends. Adjusted *p* values are reported, and *p* < 0.05 was considered statistically significant.

## 5. Conclusions

Ovotransferrin exerts in vitro candidacidal activity through a predominantly non-permeabilizing mechanism, characterized by minimal PI uptake and associated with impaired Pma1-dependent proton extrusion, disruption of K^+^ homeostasis, plasma membrane depolarization, and intracellular ATP accumulation. Mitochondrial bioenergetic activity contributes downstream to the full candidacidal response without appearing to constitute the primary antifungal target. Together, these findings support a homeostatic-threshold model in which an initially non-lytic disturbance of fungal proton and ion homeostasis becomes lethal when downstream ionic and metabolic effects exceed the compensatory capacity of the cell. To our knowledge, this study provides the first mechanistic evidence linking the candidacidal activity of ovotransferrin to disruption of fungal proton and ion homeostasis. Kal-3 and Kal-4 reproduce several key features of the response induced by the intact protein, demonstrating that γ-core-containing regions located in distinct ovotransferrin lobes can function as independent candidacidal peptides. Together with their preliminary tolerability profile, these findings support the further evaluation of ovotransferrin-derived γ-core peptides as templates for mechanism-guided antifungal development.

## Data Availability

The data presented in this study are available in the article and from the corresponding authors upon request.

## Supplementary Materials

The following supporting information can be downloaded at https://www.mdpi.com/article/10.3390/ijms27167415/s1.

## Abbreviations

The following abbreviations are used in this manuscript:

ATCC	American Type Culture Collection
CCK-8	Cell Counting Kit-8
CFU	Colony-forming unit
DiBAC_4_(3)	Bis-(1,3-dibutylbarbituric acid) trimethine oxonol
hLf	Human lactoferrin
ICP–OES	Inductively coupled plasma–optical emission spectrometry
Kal-3	Kaliocin-3
Kal-4	Kaliocin-4
Lfpep	Lactoferrin-derived membrane-permeabilizing peptide
OvoTf	Ovotransferrin (hen egg white)
PI	Propidium iodide
PierA	Piericidin A
Pma1	Plasma membrane H^+^-ATPase 1
SD	Standard deviation
SDA	Sabouraud Dextrose Agar
SDB	Sabouraud Dextrose Broth
TEA	Tetraethylammonium chloride
Tris	Tris(hydroxymethyl)aminomethane
