# Supplementary material for: Ovotransferrin and Its γ-Core-Containing Peptides Impair Pma1-Dependent Proton Homeostasis in Candida albicans
Source: Int J Mol Sci. 2026 Aug 19;27(16):7415. doi: 10.3390/ijms27167415 (PMC13513683; doi:10.3390/ijms27167415)
Supplement: Supplementary file 1 [file ijms-27-07415-s001.zip › ijms-4468777-supplementary.pdf]

A **B**

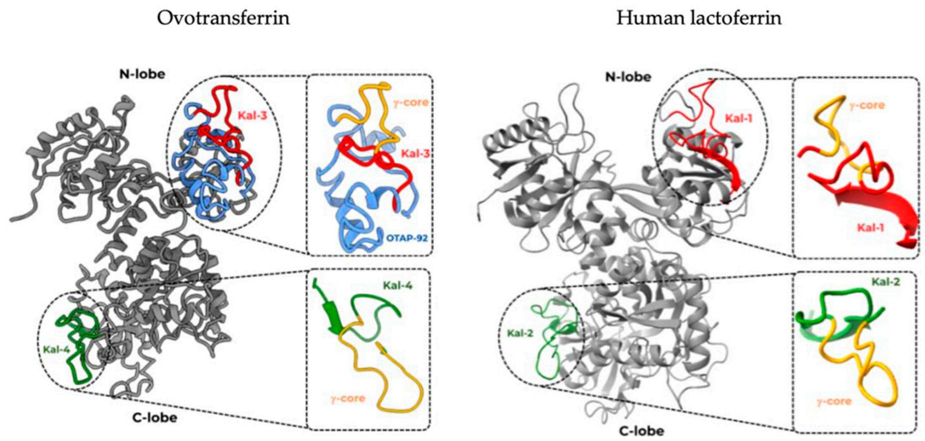

C

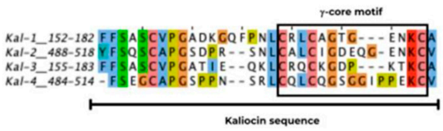

D

Protein/Peptide	Aa (No.)	m_{av}	z	H_R
**Lactoferrin**	691	76107.6	+5.68	-
Kaliocin-1	31	3190.5	+0.82	30.86
Kaliocin-2	31	3290.4	-2.18	29.56
**Ovotransferrin**	686	75756.3	-5.28	-
Kaliocin-3	29	3117.5	+2.82	22.44
Kaliocin-4	31	3119.5	-0.18	26.26

Figure S1. Comparative structural, sequence, and physicochemical analysis of γ-core-containing kaliocin regions in ovotransferrin and human lactoferrin. (**A**) Three-dimensional representation of ovotransferrin (OvoTf; PDB ID: 1AIV) showing the N-lobe and C-lobe domains. In the N-lobe, OTAP-92 is shown in blue, with Kal-3 highlighted in red and its γ-core motif in yellow within the enlarged inset. In the C-lobe, Kal-4 is shown in green, with its γ-core motif highlighted in yellow. The remaining OvoTf structure is shown in gray. (**B**) Three-dimensional representation of human lactoferrin (hLf; PDB ID: 1CB6), shown using the same orientation, structural-display settings, and color-coding as in (**A**) to facilitate direct comparison. Kal-1, the γ-core-containing region within the N-lobe kaliocin-1/lactoferricin region, is shown in red, with its γ-core motif highlighted in yellow within the enlarged inset. Kal-2, the corresponding C-lobe γ-core-containing region (kaliocin-2), is shown in green, with its γ-core motif highlighted in yellow. Enlarged views in (**A**,**B**) depict the conformations of these regions within the native full-length proteins and should not be interpreted as experimentally determined structures of the isolated peptides. (**C**) Multiple sequence alignment of the γ-core-containing kaliocin regions from human lactoferrin (Kal-1, mature hLf residues 152–182; Kal-2, mature hLf residues 488–518) and ovotransferrin (Kal-3, mature OvoTf residues 155–183; Kal-4, mature OvoTf residues 484–514). The γ-core motif is boxed, alignment gaps are represented by dashes, and conserved or chemically related residues are color-coded. (**D**) Physicochemical properties of full-length lactoferrin, full-length ovotransferrin, and the four kaliocin peptides, including peptide length, calculated average molecular mass (m_{av}), calculated net charge (z) at pH 7.0, and relative hydrophobicity (H_R); H_R was not calculated for the full-length proteins.
